# The relationship between nurse stress and professional commitment in clinical nurses: A serial multiple mediation model through resilience and coping

**DOI:** 10.1371/journal.pone.0356880

**Published:** 2026-08-24

**Authors:** Chia-En Hsieh, Li-Hua Huang, Ya-Lan Hsu, Tzu-Jung Wu, Hsiang-Chu Pai

**Affiliations:** 1 Department of Nursing, Liver transplantation center, Chung Shan Medical University Hospital, Department of Nursing, Hungkuang University, Taichung, Taiwan; 2 Department of Nursing, Chung Shan Medical University Hospital, Taichung, Taiwan; 3 Department of Nursing, Liver transplantation center, Chung Shan Medical University Hospital, Taichung, Taiwan; 4 Department of Nursing, Chung Shan Medical University Hospital, Taichung, Taiwan; 5 Department of Nursing, Chung Shan Medical University, Taichung, Taiwan; Alexandria University Faculty of Nursing, EGYPT

## Abstract

**Objectives:**

This study explored the association between work stress and professional commitment among clinical nurses, focusing on the serial multiple mediating effects of resilience and coping. Clinical nurses experience high levels of work stress that may negatively impact their professional commitment. However, the mechanisms underlying this relationship, particularly the mediating roles of resilience and coping, remain unclear.

**Materials and Methods:**

This cross-sectional study was conducted at a single medical center and involved 345 clinical nurses recruited from internal medical and surgical wards, obstetric and pediatric wards, and emergency and critical care units. The valid response rate was 97.2%. All participants were recruited from inpatient units within the same medical center, ensuring a consistent clinical setting across all departments. Descriptive, univariate, and Pearson correlation analyses were performed, and mediation effects were tested using PROCESS Model 6.

**Results:**

Nurse stress was significantly negatively associated with professional commitment (total association: c = −0.0808, p < 0.001). With resilience and coping as mediators, the direct effect remained significant (c′ = −0.0382, p = 0.0011).

**Conclusion:**

Higher nurse stress was significantly associated with lower professional commitment. Resilience and coping were significantly associated with this relationship as serial mediating factors. Given the cross-sectional design, these findings should be interpreted as associations rather than causal effects. Strengthening resilience and adaptive coping may help support nurses’ professional commitment.

## Introduction

The COVID-19 pandemic has posed unprecedented challenges to global healthcare systems. As frontline healthcare providers, nurses have shouldered substantial clinical responsibilities amid the increasing patient demands and constrained medical resources. Consequently, nursing workloads have intensified, exposing nurses to a wide range of stressors, including time pressure, organizational inadequacies, job insecurity, high work intensity, lack of recognition, sleep disturbances, work–family conflict, and fear of infection. These stressors not only undermine nurses’ physical and psychological well-being but also increase burnout, ultimately affecting care quality and patient safety [1,2]. Prolonged exposure to high-pressure work environments further weakens professional commitment and exacerbates the global nursing shortage.

Professional commitment reflects nurses’ occupational loyalty and long-term dedication to their profession, playing a vital role in workforce sustainability. It comprises three dimensions: belief in professional values, commitment to professional goals, and intention to maintain career development [3]. Previous studies have identified workplace conditions, interpersonal relationships, work–life balance, and organizational support as key determinants of professional commitment [4]. However, nurses frequently encounter adverse conditions such as workplace violence, excessive workloads, long working hours, and insufficient resources. All of these negatively affect professional commitment, job satisfaction, performance, and retention. Enhancing professional commitment through positive work attitudes, professional autonomy, adequate financial compensation, and psychological and organizational support has been shown to strengthen professional identity and occupational loyalty, thereby improving the stability and quality of nursing services [4].

Resilience refers to the ability to adapt to adversity, maintain psychological well-being, and recover from stress. Protective factors for resilience include individual psychological characteristics, external support systems, and interpersonal relationships [5]. In nursing practice, resilience is a critical psychological resource that enables nurses to remain stable under high-stress conditions [6]. The evidence suggests that nurses with higher resilience exhibit stronger internal control and experience fewer negative effects of work-related stress on their mental health and job performance [7]. Therefore, interventions aimed at enhancing resilience through training and organizational support may reduce burnout, improve professional stability, and lower turnover rates.

According to Lazarus and Folkman’s theory of stress and Coping Theory, stress occurs when environmental demands exceed an individual’s coping capacity [8]. Coping strategies may be problem-focused, targeting the stressor itself, or emotion-focused, regulating emotional responses to stress. Coping serves as a crucial buffering mechanism by adjusting cognitive and behavioral responses to mitigate the adverse effects of stress on mental and physical health [9]. In contrast, maladaptive coping strategies-such as avoidance, denial, or passive coping—can intensify psychological distress and elevate stress levels [10]. Therefore, strengthening coping abilities and promoting adaptive strategy selection are essential to improve long-term stress adjustment.

A growing body of evidence indicates that resilience and coping strategies play important roles in reducing nurses’ stress and enhancing psychological well-being [11,12]. Moreover, studies have demonstrated significant mediating effects of resilience and coping in the relationship between stress and mental health outcomes [13–15]. Although previous studies have separately examined nurse stress, resilience, coping, and professional commitment, limited evidence exists regarding the sequential psychological mechanisms through which resilience and coping jointly influence the relationship between nurse stress and professional commitment among clinical nurses. Clarifying these relationships may provide valuable insights into nurses’ psychological adaptation and career stability.

Based on this theoretical framework, the present study aimed to examine the association between work stress and nurses’ professional commitment and to investigate the multiple mediating roles of resilience and coping strategies. The following hypotheses were proposed: (1) resilience mediates the relationship between work stress and professional commitment; (2) coping mediates the relationship between work stress and professional commitment; and (3) resilience and coping act as serial mediators of this relationship. These findings have theoretical and practical insights for stress management interventions and strategies to enhance professional commitment, ultimately contributing to a more stable nursing workforce and sustainable healthcare systems.

## Methods

### Design and participants

This study employed a cross-sectional, descriptive correlational design. Data were collected through questionnaires using a one-time sampling approach rather than a longitudinal framework. The participants were nurses from the internal medical and surgical wards, obstetric and pediatric wards, and emergency and critical care units. of a medical center in central Taiwan. A convenience sampling method was used for participant recruitment. Nurse practitioners, outpatient nurses, administrative nurses, and case managers were excluded. Nurses with less than one year of experience in their current unit were also excluded. This study was approved by the Institutional Review Board (Approval Number: CS2–23171). The sample size was calculated using software, the G*Power version 3.1 [16], with the following settings: statistical test (linear multiple regression), medium effect size (0.15), significance level (0.05), statistical power (0.95). The results indicated that a minimum of 130 participants were required. Considering a 20% potential data loss rate, the final target sample size was set at 156 participants. Nevertheless, all eligible nurses available during the study period were invited to participate using convenience sampling to maximize statistical precision and improve the stability of mediation estimates. A total of 355 nurses agreed to participate, and 345 valid questionnaires were ultimately included in the final analyses, yielding a valid response rate of 97.2%. The final sample size substantially exceeded the minimum requirement and provided adequate statistical power for the serial mediation analyses conducted using PROCESS Model 6.

Prior to the study, all participants were informed of the research objectives and procedures, and anonymity in reporting the results were ensured. After signing an informed consent form, participants were asked to complete a series of questionnaires, which required approximately 20–25 minutes to complete. The recruitment period for this study was from May 1, 2024 to July 31, 2024.

### Measures

This study utilized the Nurse Stress Checklist to assess nurses’ work-related stress levels. This instrument effectively evaluates physical and psychological stress in healthcare settings, as well as stress related to personal conflicts, patient care, job performance satisfaction, and competence [17]. The Nurse Stress Checklist consists of 47 items, categorized into four dimensions: personal response (18 items), work concern (13 items), competency (11 items), and incompletion of personal arrangement (5 items), A 9-point Likert scale (0–8) was used for scoring, with higher scores indicating greater stress levels. This study adopted the Chinese version of the Nurse Stress Checklist [17], which demonstrated good internal consistency, with a Cronbach’s alpha of 0.92 [18]. In the current study, Cronbach’s alpha for the scale was 0.95, further confirming its high reliability.

To assess personal resilience, the Resilience Scale (RS) was developed by Wagnild and Young (1993) based on studies of individuals who had experienced significant life events, aiming to assess personal resilience. This scale reflects work-related resilience and includes five core dimensions: (1) purpose, (2) equanimity, (3) self-reliance, (4) perseverance, and (5) existential aloneness. The scale consists of 25 items, scored on a 7-point Likert scale ranging from “1 = strongly disagree” to “7 = strongly agree,” with higher scores indicating greater resilience. The scale has demonstrated good reliability and validity across studies, with a Cronbach’s alpha of 0.91 [19]. In the present study, the Cronbach’s alpha was 0.94, further confirming its high internal consistency and reliability.

The Brief-COPE was developed by Carver (1997) and consists of 28 items designed to assess 14 different coping strategies, including: active coping, planning, positive reframing, acceptance, humor, religion, using emotional support, using instrumental support, self-distraction, denial, venting, substance use, behavioral disengagement, and self-blame. The scale uses a 4-point Likert scale, ranging from “1 = I never do this” to “4 = I do this frequently,” with higher scores indicating more frequent use of a given coping strategy. This scale is a widely used tool for measuring coping strategies in the study population and was chosen as a core instrument. The Cronbach’s alpha values for the Brief-COPE across different coping dimensions ranged from 0.50 to 0.90 [20]. In this study, the Cronbach’s alpha was 0.89, indicating a certain level of internal consistency and suitability for the study context. In the present study, an overall Brief-COPE score was used to represent participants’ general coping tendencies within the proposed mediation framework. This approach was selected to capture the overall utilization of coping responses rather than specific coping subtypes. However, because the Brief-COPE encompasses both adaptive and maladaptive coping strategies, interpretation of the global score should be made cautiously.

The Nurses’ Professional Commitment Scale (NPCS) was developed by Lin et al. (2007). The scale consists of 19 items, scored on a 5-point Likert scale ranging from “1 = strongly disagree” to “5 = strongly agree.” The NPCS includes three dimensions: (1) nursing professional compliance, (2) involvement of nursing professionals, and (3) retention of nursing professionals. In the study by Lin et al., the Cronbach’s alpha for the scale was 0.91, demonstrating high reliability [21]. In this study, the Cronbach’s alpha was 0.944, further confirming its strong reliability and validity within the study context of the study.

### Statistical analyses

Descriptive statistics, independent sample t-tests, and one-way analysis of variance) were used to describe and compare nurses’ stress levels across different demographic characteristics, including gender, age, years of work experience, marital status, education level, and department affiliation. Pearson correlation analysis was conducted to examine the relationships among the four key study variables: (1) nurse stress, (2) resilience, (3) coping, and (4) nurse professional commitment. Data analysis was performed using IBM SPSS Statistics version 22.0 (IBM Corp, Armonk, New York, USA).

To test the study hypotheses (H1, H2, and H3), serial mediation analysis was performed using PROCESS macro Model 6 (version 4.2) developed by Hayes [22]. This method is based on the ordinary least-squares regression model and the bootstrap resampling method. The bootstrap method uses random resampling to estimate indirect effects and their 95% confidence intervals (CIs), providing more robust control for Type I errors [23]. To enhance transparency, nurse stress was specified as an independent variable, professional commitment as the dependent variable, and resilience and coping as serial mediators in the model. PROCESS Model 6 was selected because the study aimed to examine the sequential indirect effects among observed variables using a regression-based bootstrap mediation approach. Compared with SEM, PROCESS is an efficient and widely accepted method for testing serial mediation effects in cross-sectional studies with observed variables and moderate sample sizes. Serial mediation analysis was conducted using PROCESS macro Model 6, which allows the testing of both independent and sequential indirect effects. This model was selected based on a theoretical framework derived from stress and coping theory. A bootstrap resampling procedure with 2,000 iterations was applied to estimate indirect effects and their 95% confidence intervals because this method does not assume normality and provides more robust inference.

Bootstrap resampling of 2,000 iterations was used to test the indirect effects of one independent variable (nurse stress), two mediating variables (resilience and coping), and one dependent variable (nurse professional commitment). Demographic characteristics related to professional commitment were included as covariates in the analysis. The significance level was set at .05 (two-tailed test).

## Results

### Participant characteristics

A total of 345 clinical nurses participated in this study. The mean age was 32.00 ± 8.62 years (range: 22–60), and most participants were female (n = 318, 92.2%). The average length of nursing experience was 9.00 ± 8.21 years (range: 1–38), and the majority were unmarried (n = 257, 74.5%). Most participants held a bachelor’s degree (n = 301, 87.2%). Participants were recruited from medical and surgical wards (n = 183, 53.0%), obstetric and pediatric wards (n = 53, 15.4%), and emergency and critical care units (n = 109, 31.6%).

An independent-samples t test showed significant differences in nursing stress across the affiliated units (p < 0.001). Nurses working in medical and surgical wards reported the highest stress levels (Mean ± SD = 153.80 ± 57.94), followed by those in the emergency and critical care units (132.47 ± 54.41), whereas nurses in obstetric and pediatric wards reported the lowest stress levels (124.13 ± 42.81) (Table 1).

**Table 1:** Differences in Nurse stress by general characteristics (n = 345).

Variables	N = 345	Range / %	Nurse stress (Mean ± SD)	p
Age (year)	32.00 ± 8.62	22.0-60.0		0.953
20-29	186	53.9%	142.88 ± 56.76	
30-39	84	24.3%	140.36 ± 53.92	
40-49	59	17.1%	145.29 ± 58.80	
≥50	16	4.6%	139.06 ± 51.28	
Job tenure – Nursing experience (years)	9.00 ± 8.21	1.0-38.0		0.790
1- < 3	88	25.5%	140.24 ± 64.94	
3- < 6	75	21.7%	144.73 ± 49.03	
6- < 9	50	14.5%	149.26 ± 58.61	
9- < 12	40	11.6%	134.97 ± 52.89	
≥12	92	26.7%	142.45 ± 52.50	
Gender				0.435
Female	318	92.2%	143.19 ± 55.95	
Male	27	7.8%	134.41 ± 56.89	
Marital Relationship				0.956
Single	257	74.5%	141.99 ± 55.70	
Married	86	24.9%	143.93 ± 56.37	
Divorce	2	0.6%	147.00 ± 117.38	
Education level				0.367
College	20	5.8%	126.15 ± 45.42	
University	301	87.2%	143.11 ± 55.94	
Master’s and Doctoral	24	7%	148.42 ± 63.95	
Affiliated units				<0.001
Medical & surgical ward	183	53.0%	153.80 ± 57.94	
Obstetric & pediatric ward	53	15.4%	124.13 ± 42.81	
Emergency & critical care unites	109	31.6%	132.47 ± 54.41	

As shown in Table 2, the correlation analyses revealed significant associations among all study variables. Nurse stress was negatively correlated with resilience (r = −0.291, p < 0.001), coping (r = −0.296, p < 0.001), and professional commitment (r = −0.338, p < 0.001). Resilience was positively correlated with coping (r = 0.523, p < 0.001) and professional commitment (r = 0.536, p < 0.001), whereas coping was positively correlated with professional commitment (r = 0.451, p < 0.001).

**Table 2:** Pearson correlations test between nurse stress, resilience, coping, and nurse professional commitment.

Variables	Mean (SD)	1	2	3	4
1. Nurse stress	142.50 ± 55.99	1			
2. Resilience	118.39 ± 21.76	−0.291*	1		
3. Coping	77.96 ± 6.96	−0.296*	0.523*	1	
4. Nurse professional commitment	67.46 ± 13.90	−0.338*	0.536*	0.451*	1

*p < 0.01 (2-tailed).

### Serial multiple mediation analysis

**Fig 1:**
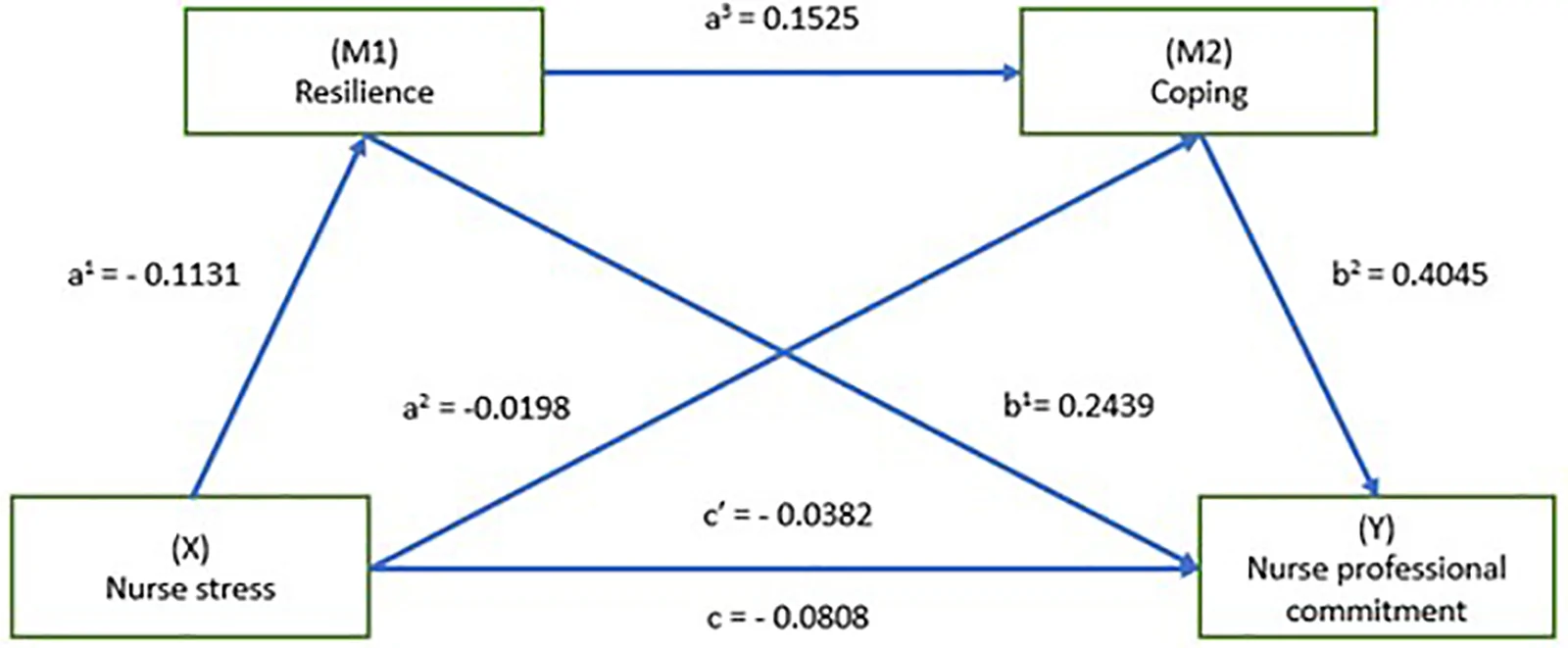
Hypothesized serial mediation model linking nurse stress and nurse professional commitment through resilience and coping as serial mediators. Nurse stress was negatively associated with resilience (a^1^ = −0.1131, p < 0.001) and coping (a^2^ = −0.0198, p = 0.001). Resilience was positively associated with coping (a^3^ = 0.1525, p < 0.001). The direct effects of mediating variables on nurse professional commitment showed that the associations involving resilience (b^1^ = 0.2439, p < 0.001) and coping (b^2^ = 0.4045, p < 0.001) were significant. The direct effect of nurse stress on nurse professional commitment was also significant (c′ = −0.0382, p = 0.001). The total effect (c = −0.0808, p < 0.001) was significantly negatively correlated.

The serial multiple mediation model (Fig 1) demonstrated that nurse stress, resilience, and coping jointly explained 35.75% of the variance in their professional commitment (R^2^ = 0.36, F = 47.31, p < 0.001). The total effect of nurse stress on professional commitment was significant and negative (c = −0.0808, SE = 0.0128, t = −6.30, p < 0.001). Nurse stress was negatively associated with resilience (a^1^ = −0.1131, SE = 0.0205, t = −5.53, p < 0.001) and coping (a^2^ = −0.0198, SE = 0.0060, t = −3.30, p = 0.001), while resilience was positively associated with coping (a^3^ = 0.1525, SE = 0.0152, t = 10.04, p < 0.001). Resilience (b^1^ = 0.2439, SE = 0.0330, t = 7.38, p < 0.001) and coping (b^2^ = 0.4045, SE = 0.1035, t = 3.91, p < 0.001) had significantly positive effects on professional commitment.

When nurse stress, resilience, and coping were entered simultaneously, the direct effect of nurse stress on professional commitment remained significant (c′ = −0.0382, SE = 0.0116, t = −3.28, p = 0.001), indicating partial mediation. After controlling for affiliated units, all indirect effects were significant (Table 3), including the single mediation by resilience (point estimate = −0.0276; 95% CI: −0.0429 to −0.0146), single mediation by coping (point estimate = −0.0080; 95% CI: −0.0150 to −0.0025), and the serial mediation via resilience and coping (point estimate = −0.0070; 95% CI: −0.0125 to −0.0027). The total indirect effect was also significant (point estimate = −0.0426; 95% CI: −0.0585 to −0.0277).

**Table 3:** Bootstrapping Indirect Effects and 95% CIs for the Final Mediational Model.

	Point Estimate	Boot SE	Boot LLCI	Boot ULCI
Effect				
A total indirect effect of X on Y	−0.0426	0.0078	−0.0585	−0.0277
Indirect effect 1: X^a^ → M1^b^ → Y^c^	−0.0276	0.0072	−0.0429	−0.0146
Indirect effect 2: X → M2^b^ → Y	−0.0080	0.0032	−0.0150	−0.0025
Indirect effect 3: X → M1 → M2 → Y	−0.0070	0.0025	−0.0124	−0.0027
Contrasts				
Model 1^e^ vs model 2^f^	−0.0196	0.0090	−0.0378	−0.0023
Model 1 vs model 3^g^	−0.0206	0.0072	−0.0364	−0.0076
Model 2 vs model 3	−0.0010	0.0036	−0.0082	0.0060

Abbreviations: CI, confidence interval; LL, lower level; UL, upper level.

a X = Nurse stress.

b M1 = Resilience.

^c^Y = Nurse professional commitment.

dM2 = Coping.

^e^Model 1 = Nurse Stress → Resilience → Nurse professional commitment.

^f^Model 2 = Nurse Stress → Coping → Nurse professional commitment.

^g^Model 3 = Nurse Stress → Resilience → Coping → Nurse professional commitment.

Comparisons between the indirect pathways indicated that the mediating effect of resilience alone was significantly stronger than that of coping alone and the serial mediation pathway. Additionally, the indirect effect of coping alone was stronger than the serial mediating effect.

## Discussion

This study examined the relationship between nurse stress and professional commitment, as well as the mediating roles of resilience and coping among clinical nurses. The findings demonstrated that nurse stress was significantly and negatively associated with professional commitment, both directly and indirectly through the mediating effects of resilience and coping strategies. These findings should be interpreted as associative rather than causal because of the cross-sectional design. Additionally, the results confirmed that resilience was positively associated with coping, further contributing to the serial-multiple mediation effect in this relationship.

Numerous studies have indicated that clinical nurses working in high-stress environments experience a decline in professional commitment, including professional identity, job engagement, and retention intentions [24], ultimately leading to increased staff turnover. According to statistics from Taiwan’s Ministry of Health and Welfare, the nursing vacancy rate will increase from 4.48% in 2018 (pre-pandemic) to 6.53% in 2022, whereas the nurse turnover rate will increase from 10.04% in 2018 to 11.7% in 2022, reaching 12.61% in 2023 [25]. This trend suggests that despite the easing of the pandemic, issues related to nurse attrition and job vacancies have not improved and have even worsened. Consequently, many hospitals have been forced to reduce the number of available hospital beds and disrupting patient admissions and surgical scheduling. This vicious cycle negatively impacts nurses’ job autonomy, prompting many highly motivated or career-competitive nurses to transition to other health-related industries, such as insurance, health supplements, pharmaceuticals, and medical equipment sales, leaving behind clinical nursing roles in major hospitals [26]. Prolonged exposure to high-stress work environments not only exacerbates this trend but also has a profound negative impact on nurses’ professional commitment. Therefore, in addition to reducing workplace stress, providing support for resilience and coping strategies is crucial for addressing this issue.

Hypothesis 1 proposed that resilience mediates the relationship between nurse stress and professional commitment. The findings of this study supported this hypothesis, indicating that resilience serves as a key psychological mediator between nurse stress and professional commitment. Resilience, as a form of psychological flexibility, enables individuals to adapt to and recover from adversity and stress. Previous research has indicated that higher resilience levels are associated with reduced emotional exhaustion and stress, as well as increased work engagement and job satisfaction [27]. Resilience training enhances nurses’ adaptability, and reduces anxiety, depression, and burnout [28]. Specific resilience-building strategies include: mindfulness and relaxation training, psychoeducation, emotion regulation techniques, cognitive strategies, problem-solving skills, and strengthening of internal and external support resources. These strategies have demonstrated moderate positive effects in previous studies [29]. However, nursing resilience in nursing is a self-regulatory process that involving both internal factors (e.g., cognitive appraisal and adaptive thinking) and external resources (e.g., workplace support and clinical administrative oversight) [30]. When organizational support is insufficient, nurses’ self-regulatory capacity is constrained, potentially leading to negative consequences or an imbalance between stress and professional commitment. Therefore, governments and healthcare institutions should implement supportive policies, such as: optimizing nurse-to-patient ratios, effective workload management, enhancing workplace support systems, and providing continuous education and professional development. These measures will help nurses leverage resilience to maintain their professional commitment in high-stress environments.

Hypothesis 2 proposed that coping would mediate the relationship between nurse stress and professional commitment. The findings supported this hypothesis, demonstrating that coping functions as an important mediator between nurse stress and professional commitment. Coping refers to an individual’s cognitive and behavioral processes used to evaluate events -including external and internal demands, challenges, and conflicts -while seeking internal and external support and resources to effectively manage stress [8]. This process is dynamic and is continuously adjusted based on situational changes and individual psychological states. Recent research on COVID-19-related stress has indicated that nurses can adapt to workplace stress by employing strategies such as acceptance, religious beliefs, planning, and positive reframing, while integrating ethical and mindfulness-based approaches to enhance infection prevention training and professional competency [31,32]. Additionally, psychosocial coping strategies, communication with family members, and helping others are considered flexible coping mechanisms commonly used by nurses. Studies have shown that nurses with better coping abilities experience lower levels of stress [33]. However, some studies have revealed that certain nurses exhibited maladaptive coping tendencies during the pandemic, such as fearing the risk of infecting family members or regretting their career choice [32]. Psychological intervention techniques, including cognitive processing therapy, emotional freedom techniques, prolonged exposure therapy, eye movement desensitization, and reprocessing, may offer effective support when nurses face negative coping responses. These techniques have been shown to reduce the psychological burdens associated with pandemic-related stress, assist nurses in developing adaptive coping strategies, and enable them to gradually reintegrate into society and return to their professional nursing roles through continuous adaptation and accumulated experience [34]. Therefore, to ensure nurses’ psychological well-being, healthcare institutions and related support systems should provide necessary assistance and resources to enhance their coping abilities, ultimately improving job stability and strengthening professional commitment.

Hypothesis 3 proposed that resilience and coping operate as serial mediators in the relationship between nurse stress and professional commitment. These findings supported our hypothesis and demonstrated a significant serial mediation pathway through resilience and coping. The results indicate that higher levels of nurse stress significantly reduce professional commitment, and resilience and coping strategies played crucial mediating roles in potentially buffering this negative association. As a core indicator of psychological resilience, it helps nurses maintain emotional stability and professional engagement in high-stress environments. Additionally, appropriate coping strategies – particularly problem-focused coping – enable nurses to effectively manage work challenges, thereby reducing the adverse effects of stress on professional commitment. Despite the gradual decline during the COVID-19 pandemic, the nursing profession continues to face workforce shortages and increasing vacancy rates. This trend suggests that beyond demographic challenges such as low birth rates, the nursing work environment requires more proactive policies and resource support. By enhancing professional knowledge and cultivating a rational yet optimistic mindset, nurses can improve their ability to manage stress, thereby reinforcing their professional identity and fostering greater enthusiasm for their work. Overall, strengthening nurses’ psychological well-being, professional commitment, and stress management skills will contribute to greater long-term retention, stabilizing the nursing workforce, and ultimately ensuring high-quality healthcare services.

Despite facing considerable workplace challenges, nurses continued to demonstrate strong professional commitment and dedication to patient care, particularly as frontline healthcare providers and first responders. The findings indicated that respondents experienced moderate to high levels of perceived work stress, accompanied by relatively low psychological resilience, while maintaining moderate levels of work engagement and self-efficacy [35]. Despite these stressors, many nurses actively developed personal coping strategies and adaptive mechanisms to manage occupational demands [36]. Effective stress adjustment strategies, including coping abilities and psychological hardiness, appeared to strengthen nurses’ commitment to their professional practice and support their continued engagement in clinical responsibilities [37]. These findings highlight the importance of enhancing resilience-building and stress-management interventions to sustain nurses’ professional commitment and overall well-being in demanding healthcare environments. Although resilience and coping partially mediated the relationship between nurse stress and professional commitment, the direct effect of stress remained significant, suggesting that additional organizational factors may also influence professional commitment. Factors such as staffing adequacy, workload intensity, leadership support, workplace culture, scheduling flexibility, and institutional support systems may substantially affect nurses’ professional engagement and retention intentions. Therefore, future studies should incorporate these organizational variables to develop a more comprehensive explanatory framework regarding the factors influencing nurses’ professional commitment.

### Limitations

Due to the cross-sectional design, causal relationships cannot be established, and the findings should be interpreted as associations rather than causal effects. In addition, although the a priori sample size calculation was based on a multiple regression framework rather than a specific serial mediation model, the final sample substantially exceeded the estimated minimum requirement, thereby enhancing the precision and stability of the mediation estimates.

Participants were recruited using convenience sampling from a single large medical center in central Taiwan, which may limit the generalizability of the findings. Compared with smaller community hospitals, large medical centers may differ in terms of patient acuity, workload intensity, staffing patterns, organizational resources, and workplace support systems. These institutional characteristics may influence nurses’ stress experiences, coping processes, resilience, and professional commitment.

Furthermore, all study variables were assessed using self-reported questionnaires, which may introduce recall bias or social desirability bias. The present study employed a global coping score derived from the Brief-COPE. Because the Brief-COPE includes both adaptive and maladaptive coping strategies, the overall score may have obscured the distinct relationships between specific coping dimensions and professional commitment. Future research is encouraged to examine Brief-COPE subdimensions separately to clarify their unique mediating roles and enhance the precision of interpretation.

Finally, longitudinal and multi-center studies are warranted to further verify the proposed pathways and enhance the generalizability and applicability of the findings across diverse healthcare settings.

## Conclusion

Nurse stress was significantly associated with lower professional commitment among clinical nurses, with resilience and coping being significantly associated mediating factors within the observed relationships. These findings highlight the potential importance of psychological resources in understanding how nurses respond to workplace stress. However, given the cross-sectional design and single-center setting, the results should be interpreted with caution as associative rather than causal. Future longitudinal and multi-center studies are warranted to further validate these relationships and explore the causal pathways. Interventions aimed at enhancing resilience and adaptive coping may be beneficial for supporting nurses’ professional commitment.
