# Supplementary material for: The relationship between nurse stress and professional commitment in clinical nurses: A serial multiple mediation model through resilience and coping
Source: PLoS One. 2026 Aug 24;21(8):e0356880. doi: 10.1371/journal.pone.0356880 (PMC13502600; doi:10.1371/journal.pone.0356880)
Supplement: Supplementary file 1 [file pone.0356880.s001.pdf]

PLOS One

The relationship between nurse stress and professional commitment in clinical nurses: A serial multiple mediation model through resilience and coping

--Manuscript Draft--

**Manuscript Number:**	PONE-D-26-11739
**Article Type:**	Research Article
**Full Title:**	The relationship between nurse stress and professional commitment in clinical nurses: A serial multiple mediation model through resilience and coping
**Short Title:**	Nurse stress, resilience & commitment
**Corresponding Author:**	Chia-En Hsieh, Ph.D.<br>Chung Shan Medical University Hospital<br>Taichung, --- Select One --- TAIWAN
**Keywords:**	nurse stress, resilience, coping, professional commitment
**Abstract:**	Objectives: This study explores the association between work stress and professional commitment in clinical nurses, focusing on the serial multiple mediating effects of resilience and coping. Clinical nurses experience high work stress, which may negatively impact their professional commitment. However, the mechanisms underlying this relationship, particularly the mediating roles of resilience and coping, remain unclear.<br>Materials and Methods: A cross-sectional study was conducted at a medical center, involving 345 nurses from internal medicine, surgery, obstetrics, and pediatrics departments. Descriptive, univariate, and Pearson correlation analyses were performed, and mediation effects were tested using PROCESS Model 6.<br>Results: Nursing stress had a significant negative total effect on professional commitment ($c = -0.0808$, $p < .001$). With resilience and coping as mediators, the direct effect remained significant ($c' = -0.0382$, $p = .0011$).<br>Conclusion: Work stress negatively affects professional commitment, both directly and indirectly. Strengthening resilience and coping may help mitigate this impact.
**Order of Authors:**	Chia-En Hsieh, Ph.D.
	Li-Hua Huang
	Ya-Lan Hsu
	Tzu-Jung Wu
	Hsiang-Chu Pai
**Additional Information:**	
**Question**	**Response**
**Financial Disclosure**<br><br>Enter a financial disclosure statement that describes the sources of funding for the work included in this submission. Review the submission guidelines for detailed requirements. View published research articles from *PLOS ONE* for specific examples.<br><br>This statement is required for submission and **will appear in the published article** if the submission is accepted. Please make	Yes

sure it is accurate.<br><br>**Funded studies**<br>Enter a statement with the following details:<br>• Initials of the authors who received each award<br>• Grant numbers awarded to each author<br>• The full name of each funder<br>• URL of each funder website<br>• Did the sponsors or funders play any role in the study design, data collection and analysis, decision to publish, or preparation of the manuscript?<br><br>Did you receive funding for this work?	
Please add funding details.<br>as follow-up to "**Financial Disclosure**<br><br>Enter a financial disclosure statement that describes the sources of funding for the work included in this submission. Review the submission guidelines for detailed requirements. View published research articles from *PLOS ONE* for specific examples.<br><br>This statement is required for submission and **will appear in the published article** if the submission is accepted. Please make sure it is accurate.<br><br>**Funded studies**<br>Enter a statement with the following details:<br>• Initials of the authors who received each award<br>• Grant numbers awarded to each author<br>• The full name of each funder<br>• URL of each funder website<br>• Did the sponsors or funders play any role in the study design, data collection and analysis, decision to publish, or preparation of the manuscript?<br><br>Did you receive funding for this work?"	Author Chia-En Hsieh received a grant (CSH-2024-A-040) from the Chung Shan Medical University Hospital Foundation. The funding entities had no role in the study's design, the collection, analysis, the interpretation of data, or the writing of the manuscript.
Please select the country of your main research funder (please select carefully	TAIWAN - TW

as in some cases this is used in fee calculation).<br>as follow-up to "**Financial Disclosure**<br><br>Enter a financial disclosure statement that describes the sources of funding for the work included in this submission. Review the submission guidelines for detailed requirements. View published research articles from *PLOS ONE* for specific examples.<br><br>This statement is required for submission and **will appear in the published article** if the submission is accepted. Please make sure it is accurate.<br><br>**Funded studies**<br>Enter a statement with the following details:<br>• Initials of the authors who received each award<br>• Grant numbers awarded to each author<br>• The full name of each funder<br>• URL of each funder website<br>• Did the sponsors or funders play any role in the study design, data collection and analysis, decision to publish, or preparation of the manuscript?<br><br>Did you receive funding for this work?"	
**Competing Interests**<br><br>Use the instructions below to enter a competing interest statement for this submission. On behalf of all authors, disclose any competing interests that could be perceived to bias this work—acknowledging all financial support and any other relevant financial or non-financial competing interests.<br><br>This statement is required for submission and **will appear in the published article** if the submission is accepted. Please make sure it is accurate and that any funding sources listed in your Funding Information later in the submission form are also	The authors have declared that no competing interests exist

declared in your Financial Disclosure statement.<br><br>View published research articles from *PLOS ONE* for specific examples.<br><br>**NO authors have competing interests**<br><br>Enter: *The authors have declared that no competing interests exist.*<br><br>**Authors with competing interests**<br><br>Enter competing interest details beginning with this statement:<br><br>*I have read the journal's policy and the authors of this manuscript have the following competing interests: [insert competing interests here]*<br><br>* typeset	
**Ethics Statement**<br><br>Enter an ethics statement for this submission. This statement is required if the study involved:<br><br>• Human participants<br>• Human specimens or tissue<br>• Vertebrate animals or cephalopods<br>• Vertebrate embryos or tissues<br>• Field research<br><br>Write "N/A" if the submission does not require an ethics statement.<br><br>General guidance is provided below. Consult the submission guidelines for detailed instructions. **Make sure that all information entered here is included in the Methods section of the manuscript.**	The study was approved by the Institutional Review Board of CS2-23171. All authors approved the final version for submission.

**Format for specific study types**<br><br>**Human Subject Research (involving human participants and/or tissue)**<br>• Give the name of the institutional review board or ethics committee that approved the study<br>• Include the approval number and/or a statement indicating approval of this research<br>• Indicate the form of consent obtained (written/oral) or the reason that consent was not obtained (e.g. the data were analyzed anonymously)<br><br>**Animal Research (involving vertebrate animals, embryos or tissues)**<br>• Provide the name of the Institutional Animal Care and Use Committee (IACUC) or other relevant ethics board that reviewed the study protocol, and indicate whether they approved this research or granted a formal waiver of ethical approval<br>• Include an approval number if one was obtained<br>• If the study involved *non-human primates*, add *additional details* about animal welfare and steps taken to ameliorate suffering<br>• If anesthesia, euthanasia, or any kind of animal sacrifice is part of the study, include briefly which substances and/or methods were applied<br><br>**Field Research**<br><br>Include the following details if this study involves the collection of plant, animal, or other materials from a natural setting:<br>• Field permit number<br>• Name of the institution or relevant body that granted permission	
**Data Availability**<br><br>Authors are required to make all data underlying the findings described fully available, without restriction, and from the time of publication. PLOS allows rare exceptions to address legal and ethical concerns. See the PLOS Data Policy and FAQ for detailed information.	No - some restrictions will apply

Powered by Editorial Manager® and ProduXion Manager® from Aries Systems Corporation

A Data Availability Statement describing where the data can be found is required at submission. Your answers to this question constitute the Data Availability Statement and **will be published in the article**, if accepted.<br><br>**Important:** Stating 'data available on request from the author' is not sufficient. If your data are only available upon request, select 'No' for the first question and explain your exceptional situation in the text box.<br><br>Do the authors confirm that all data underlying the findings described in their manuscript are fully available without restriction?	
**Describe where the data may be found in full sentences. If you are copying our sample text, replace any instances of XXX with the appropriate details.**<br><br>• If the data are **held or will be held in a public repository**, include URLs, accession numbers or DOIs. If this information will only be available after acceptance, indicate this by ticking the box below. For example: *All XXX files are available from the XXX database (accession number(s) XXX, XXX.).*<br>• If the data are all contained **within the manuscript and/or Supporting Information files**, enter the following: *All relevant data are within the manuscript and its Supporting Information files.*<br>• If neither of these applies but you are able to provide **details of access elsewhere**, with or without limitations, please do so. For example:<br><br>*Data cannot be shared publicly because of [XXX]. Data are available from the XXX Institutional Data Access / Ethics Committee (contact via XXX) for researchers who meet the criteria for access to confidential data.*<br><br>*The data underlying the results presented in the study are available from (include the name of the third party*	Ethical restrictions prohibit the authors from making the data publicly available in order to protect the confidentiality and privacy of participants. However, interested researchers can contact the corresponding author to discuss possibilities for data access. Access to data can be made by completion of appropriate forms which require Institution Review Board and further information for data access is available by chiaen69918@gmail.com who will process the application and forward to the liver transplantation group.

and contact information or URL).<br>• This text is appropriate if the data are owned by a third party and authors do not have permission to share the data.<br><br>* typeset	
Additional data availability information:	Tick here if the URLs/accession numbers/DOIs will be available only after acceptance of the manuscript for publication so that we can ensure their inclusion before publication.

Manuscript Click here to access/download;Manuscript;main.docx

Title page

Title: The relationship between nurse stress and professional commitment in clinical nurses: A serial multiple mediation model through resilience and coping

Short running title: Nurse stress, resilience & commitment

Authors: Name / E-mail

Chia-En Hsieh[1], PhD, RN, NP, Assistant Professor, chiaen69918@gmail.com；

Li-Hua Huang[2], MSN, RN, csha978@csh.org.tw；

Ya-Lan Hsu[3], MSN, RN, NP, nsi151066@gmail.com；

Tzu-Jung Wu[4], MSN, RN, cshx367@csh.org.tw；

Hsiang-Chu Pai[5], PhD, RN, Professor, pai55215@csmu.edu.tw

Affiliation / institutions:

1. Nurse Practitioner, Department of Nursing, Liver transplantation center, Chung Shan Medical University Hospital, Taichung, Taiwan, and Assistant Professor, Department of Nursing, Hungkuang University, Taichung, Taiwan

2. Vice Director, Department of Nursing, Chung Shan Medical University Hospital, Taichung, Taiwan

3. Nurse Practitioner, Department of Nursing, Liver transplantation center, Chung Shan Medical University Hospital, Taichung, Taiwan.

4. Vice Superintendent, Department of Nursing, Chung Shan Medical University Hospital, Taichung, Taiwan

5. Professor, Department of Nursing, Chung Shan Medical University, Taichung, Taiwan.

Author`s Contributions

Conceptualization, and methodology: Chia-En Hsieh, Tzu-Jung Wu, Hsiang-Chu Pai

Data curation, and investigation: Ya-Lan Hsu

Project administration, and resources: Li-Hua Huang

Software, Formal analysis, Funding acquisition: Chia-En Hsieh

Supervision, validation, and visualization: Hsiang-Chu Pai, Tzu-Jung Wu

Roles/Writing - original draft: Chia-En Hsieh, Li-Hua Huang, Ya-Lan Hsu

Writing - review & editing: Chia-En Hsieh, Hsiang-Chu Pai

All authors read and approved the final manuscript.

Corresponding and reprint requested to:

1. Hsiang-Chu Pai, RN, PhD, Professor

Professor, Department of Nursing, Chung Shan Medical University, Taichung, Taiwan.

No. 110, Sec. 1, Jianguo N. Rd., South Dist., Taichung City 40201, Taiwan

E-mail: pai55215@csmu.edu.tw

2. Tzu-Jung Wu, RN, MSN

Vice Superintendent, Department of Nursing, Chung Shan Medical University Hospital, Taichung, Taiwan

No. 110, Sec. 1, Jianguo N. Rd., South Dist., Taichung City 40201, Taiwan

E-mail: cshx367@csh.org.tw

Acknowledgements

Sincere gratitude to the head nurses of medical, surgical, obstetric, pediatric, and critical care nursing units. They also encourage the nursing staff in these units to participate in completing this research questionnaire.

Conflict of interest statement:

The authors declare that they have no conflicts of interest statement. The study was approved by the Institutional Review Board of CS2-23171. All authors approved the final version for submission.

Financial support statement:

Author Chia-En Hsieh received a grant (CSH-2024-A-040) from the Chung Shan Medical University Hospital Foundation. The funding entities had no role in the study's design, the collection, analysis, the interpretation of data, or the writing of the manuscript.

Declaration of generative AI in scientific writing

The author uses artificial intelligence technology to enhance language and readability.

The relationship between nurse stress and professional commitment in clinical nurses: A serial multiple mediation model through resilience and coping

Abstract

Objectives: This study explores the association between work stress and professional commitment in clinical nurses, focusing on the serial multiple mediating effects of resilience and coping. Clinical nurses experience high work stress, which may negatively impact their professional commitment. However, the mechanisms underlying this relationship, particularly the mediating roles of resilience and coping, remain unclear.

Materials and Methods: A cross-sectional study was conducted at a medical center, involving 345 nurses from internal medicine, surgery, obstetrics, and pediatrics departments. Descriptive, univariate, and Pearson correlation analyses were performed, and mediation effects were tested using PROCESS Model 6.

Results: Nursing stress had a significant negative total effect on professional commitment ($c = -0.0808$, $p < .001$). With resilience and coping as mediators, the direct effect remained significant ($c' = -0.0382$, $p = .0011$).

Conclusion: Work stress negatively affects professional commitment, both directly and indirectly. Strengthening resilience and coping may help mitigate this impact.

Keywords: nurse stress, resilience, coping, professional commitment

Introduction

The COVID-19 pandemic has posed unprecedented challenges to global healthcare systems. As frontline healthcare providers, nurses have shouldered substantial clinical responsibilities amid surging patient demands and constrained medical resources. Consequently, nursing workloads have intensified, exposing nurses to a wide range of stressors, including time pressure, organizational inadequacies, job insecurity, high work intensity, lack of recognition, sleep disturbances, work–family conflict, and fear of infection. These stressors not only undermine nurses' physical and psychological well-being but also increase burnout, adversely affecting care quality and patient safety [1, 2]. Prolonged exposure to high-pressure work environments further weakens professional commitment and exacerbates the global nursing shortage.

Professional commitment reflects nurses' occupational loyalty and long-term dedication to their profession, playing a vital role in workforce sustainability. It comprises three dimensions: belief in professional values, commitment to professional goals, and intention to maintain career development [3]. Previous studies have identified workplace conditions, interpersonal relationships, work–life balance, and organizational support as key determinants of professional commitment [4]. However, nurses frequently encounter adverse conditions such as workplace violence, excessive workloads, long working hours, and insufficient resources, all of which negatively affect professional commitment, job satisfaction, performance, and retention. Enhancing professional commitment through positive work attitudes, professional autonomy, adequate financial compensation, and psychological and organizational support has been shown to strengthen

professional identity and occupational loyalty, thereby improving the stability and quality of nursing services [4].

Resilience refers to the ability to adapt to adversity, maintain psychological well-being, and recover from stress. Protective factors for resilience include individual psychological characteristics, external support systems, and interpersonal relationships [5]. In nursing practice, resilience functions as a critical psychological resource that enables nurses to remain stable under high-stress conditions [6]. Evidence suggests that nurses with higher resilience exhibit stronger internal control and experience fewer negative effects of work-related stress on mental health and job performance [7]. Interventions aimed at enhancing resilience through training and organizational support may therefore reduce burnout, improve professional stability, and lower turnover rates.

According to Lazarus and Folkman's Stress and Coping Theory, stress occurs when environmental demands exceed an individual's coping capacity [8]. Coping strategies may be problem-focused, targeting the stressor itself, or emotion-focused, regulating emotional responses to stress. Coping serves as a crucial buffering mechanism by adjusting cognitive and behavioral responses to mitigate the adverse effects of stress on mental and physical health [9]. In contrast, maladaptive coping strategies-such as avoidance, denial, or passive coping—can intensify psychological distress and elevate stress levels [10]. Strengthening coping abilities and promoting adaptive strategy selection are therefore essential for improving long-term stress adjustment.

A growing body of evidence indicates that resilience and coping strategies play important roles

in reducing nurses' stress and enhancing psychological well-being [11,12]. Moreover, studies have demonstrated significant mediating effects of resilience and coping in the relationship between stress and mental health outcomes [13-15]. However, the combined and sequential mechanisms through which work stress, resilience, coping strategies, and professional commitment interact among nurses remain insufficiently explored. Clarifying these relationships may provide valuable insights into nurses' psychological adaptation and career stability.

Based on this theoretical framework, the present study aims to examine the impact of work stress on nurses' professional commitment and to investigate the multiple mediating roles of resilience and coping strategies. The following hypotheses are proposed: (1) resilience mediates the relationship between work stress and professional commitment; (2) coping mediates the relationship between work stress and professional commitment; and (3) resilience and coping act as serial mediators in this relationship. The findings are expected to offer theoretical and practical implications for stress management interventions and strategies to enhance professional commitment, ultimately contributing to a more stable nursing workforce and sustainable healthcare systems.

Methods

Design and Participants

This study adopted a descriptive correlational research design using a cross-sectional approach and collected data through questionnaire surveys. The participants were nurses from the internal

medicine, surgery, obstetrics and gynecology, and pediatrics departments of a medical center in central Taiwan. A convenience sampling method was used for participant recruitment. Nurse practitioners, outpatient nurses, administrative nurses, and case managers were excluded. Nurses with less than one year of experience in their current unit were also excluded. This study was approved by the Institutional Review Board (Approval Number: CS2-23171). The sample size was calculated using the G*Power 3.1 software [16], with the following settings: statistical test (linear multiple regression), medium effect size (0.15), significance level (0.05), statistical power (0.95). The results indicated that a minimum of 130 participants were required. Considering a 20% potential data loss rate, the final target sample size was set at 156 participants. A total of 355 eligible participants were successfully recruited, achieving a 100% response rate. Among them, 10 questionnaires were incomplete and therefore excluded, resulting in a final valid sample of 345 participants. Prior to the study, all participants were informed of the research objectives and procedures, and anonymity in reporting the results was assured. After signing an informed consent form, participants were asked to complete a series of questionnaires, which took approximately 20–25 minutes to complete. The recruitment period for this study was from May 1, 2024 to July 31, 2024.

Measures

This study utilized the Nurse Stress Checklist to assess nurses' work-related stress levels. This instrument effectively evaluates physical and psychological stress in healthcare settings, as well as stress related to personal conflicts, patient care, job performance satisfaction, and competence [17].

The Nurse Stress Checklist consists of 47 items, categorized into four dimensions: personal response (18 items), work concern (13 items), competency (11 items), incompletion of personal arrangement (5 items), A 9-point Likert scale (0–8) was used for scoring, with higher scores indicating greater stress levels. This study adopted the Chinese version of the Nurse Stress Checklist,[17] which demonstrated good internal consistency, with a Cronbach's alpha of 0.92 [18]. In the current study, the scale's Cronbach's alpha was 0.95, further confirming its high reliability.

The Resilience Scale (RS) was developed by Wagnild and Young (1993) based on studies of individuals who had experienced significant life events, aiming to assess personal resilience. This scale reflects work-related resilience and includes five core dimensions: (1) purpose, (2) equanimity, (3) self-reliance, (4) perseverance, (5) existential aloneness. The scale consists of 25 items, scored on a 7-point Likert scale ranging from "1 = strongly disagree" to "7 = strongly agree," with higher scores indicating greater resilience. The scale demonstrated good reliability and validity across studies, with a Cronbach's alpha of 0.91 [19]. In the present study, the Cronbach's alpha was 0.94, further confirming its high internal consistency and reliability.

The Brief-COPE was developed by Carver (1997) and consists of 28 items designed to assess 14 different coping strategies, including: Active coping, planning, positive reframing, acceptance, humor, religion, using emotional support, using instrumental support, self-distraction, denial, venting, substance use, behavioral disengagement, and self-blame. The scale uses a 4-point Likert scale, ranging from "1 = I never do this" to "4 = I do this frequently," with higher scores indicating more frequent use of a given coping strategy. This scale is a widely used tool for measuring coping

strategies among the study population and was chosen as a core instrument. The Cronbach's alpha of the Brief-COPE across different coping dimensions ranged from 0.50 to 0.90 [20]. In this study, the Cronbach's alpha was 0.89, indicating a certain level of internal consistency and suitability for the study context.

The Nurses' Professional Commitment Scale (NPCS) was developed by Lin et al. (2007) to assess nurses' commitment to their profession. The scale consists of 19 items, scored on a 5-point Likert scale ranging from "1 = strongly disagree" to "5 = strongly agree." The NPCS includes three dimensions: (1) nursing professional compliance, (2) involvement of nursing professionals, (3) retention of nursing professionals. In Lin et al.'s study, the Cronbach's alpha for the scale was 0.91, demonstrating high reliability [21]. In this study, the Cronbach's alpha was 0.944, further confirming its strong reliability and validity within the study context.

Statistical analyses

Descriptive statistics, independent sample t-tests, and one-way analysis of variance) re used to describe and compare nurses' stress levels across different demographic characteristics, including gender, age, years of work experience, marital status, education level, and department affiliation. Pearson correlation analysis was conducted to examine the relationships among the four key study variables: (1) nurse stress, (2) resilience, (3) coping, (4) nurse professional commitment. Data analysis was performed using IBM SPSS Statistics version 22.0 (IBM Corp, Armonk, New York). To test the study hypotheses (H1, H2, H3), a serial mediation model (Model 6) from the PROCESS macro (version 4.2) by Hayes was used [22]. This method is based on the ordinary least-squares

regression model and the bootstrap method. The bootstrap method uses random resampling to estimate indirect effects and their 95% confidence intervals (CIs), providing more robust control for Type I errors [23]. A bootstrap resampling of 2,000 iterations was used to test the indirect effects of one independent variable (nurse stress), two mediating variables (resilience and coping), and one dependent variable (nurse professional commitment). Additionally, demographic characteristics related to professional commitment were included as covariates in the analysis. The significance level was set at .05 (two-tailed test).

Results

Participant characteristics

A total of 345 clinical nurses participated in this study. The mean age was 32.00 ± 8.62 years (range: 22–60), and most participants were female (n = 318, 92.2%). The average length of nursing experience was 9.00 ± 8.21 years (range: 1–38), and the majority were unmarried (n = 257, 74.5%). Most participants held a bachelor's degree (n = 301, 87.2%). Participants were recruited from medical and surgical wards (n = 183, 53.0%), obstetric and pediatric wards (n = 53, 15.4%), and emergency and critical care units (n = 109, 31.6%).

An independent-samples t test showed significant differences in nursing stress across affiliated units ($p < 0.001$). Nurses working in medical and surgical wards reported the highest stress levels (Mean ± SD = 153.80 ± 57.94), followed by those in emergency and critical care units (132.47 ± 54.41), whereas nurses in obstetric and pediatric wards reported the lowest stress levels (124.13 ±

42.81) (Table 1).

As shown in Table 2, correlation analyses revealed significant associations among all study variables. Nurse stress was negatively correlated with resilience ($r = -0.291$, $p < 0.001$), coping ($r = -0.296$, $p < 0.001$), and professional commitment ($r = -0.338$, $p < 0.001$). In contrast, resilience was positively correlated with coping ($r = 0.523$, $p < 0.001$) and professional commitment ($r = 0.536$, $p < 0.001$), and coping was positively correlated with professional commitment ($r = 0.451$, $p < 0.001$).

Serial multiple mediation analysis

The serial multiple mediation model (Figure 1) demonstrated that nurse stress, resilience, and coping jointly explained 35.75% of the variance in professional commitment ($R^2 = 0.36$, $F = 47.31$, $p < 0.001$). The total effect of nurse stress on professional commitment was significant and negative ($c = -0.0808$, $SE = 0.0128$, $t = -6.30$, $p < 0.001$). Nurse stress was negatively associated with resilience ($a^1 = -0.1131$, $SE = 0.0205$, $t = -5.53$, $p < 0.001$) and coping ($a^2 = -0.0198$, $SE = 0.0060$, $t = -3.30$, $p = 0.001$), while resilience was positively associated with coping ($a^3 = 0.1525$, $SE = 0.0152$, $t = 10.04$, $p < 0.001$). Both resilience ($b^1 = 0.2439$, $SE = 0.0330$, $t = 7.38$, $p < 0.001$) and coping ($b^2 = 0.4045$, $SE = 0.1035$, $t = 3.91$, $p < 0.001$) had significant positive effects on professional commitment.

When nurse stress, resilience, and coping were entered simultaneously, the direct effect of nurse stress on professional commitment remained significant ($c' = -0.0382$, $SE = 0.0116$, $t = -3.28$, $p = 0.001$), indicating partial mediation. After controlling for affiliated units, all indirect

effects were significant (Table 3), including the single mediation by resilience (point estimate = −0.0276; 95% CI: −0.0429 to −0.0146), single mediation by coping (point estimate = −0.0080; 95% CI: −0.0150 to −0.0025), and the serial mediation via resilience and coping (point estimate = −0.0070; 95% CI: −0.0125 to −0.0027). The total indirect effect was also significant (point estimate = −0.0426; 95% CI: −0.0585 to −0.0277).

Comparisons among indirect pathways indicated that the mediating effect of resilience alone was significantly stronger than that of coping alone and the serial mediation pathway. Additionally, the indirect effect through coping alone was stronger than the serial mediation effect.

Discussion

This study examined the relationship between nurse stress and professional commitment, as well as the mediating roles of resilience and coping among clinical nurses. The findings demonstrated that nurse stress was significantly and negatively associated with professional commitment, both directly and indirectly through the mediating effects of resilience and coping strategies. Additionally, the results confirmed that resilience influences coping, further contributing to the serial-multiple mediation effect in this relationship.

Numerous studies have indicated that clinical nurses working in high-stress environments experience a decline in professional commitment, including professional identity, job engagement, and retention intentions [24], ultimately leading to increased staff turnover. According to statistics from Taiwan’s Ministry of Health and Welfare, the nursing vacancy rate rose from 4.48% in 2018

(pre-pandemic) to 6.53% in 2022, while the nurse turnover rate increased from 10.04% in 2018 to 11.7% in 2022, reaching 12.61% in 2023 [25]. This trend suggests that despite the easing of the pandemic, issues related to nurse attrition and job vacancies have not improved and have even worsened. As a result, many hospitals have been forced to reduce the number of available hospital beds, disrupting patient admissions and surgical scheduling. This vicious cycle negatively impacts nurses' job autonomy, prompting many highly motivated or career-competitive nurses to transition to other health-related industries, such as insurance, health supplements, pharmaceuticals, or medical equipment sales, leaving behind clinical nursing roles in major hospitals [26]. Prolonged exposure to high-stress work environments not only exacerbates this trend but also has a profound negative impact on nurses' professional commitment. Therefore, in addition to reducing workplace stress, providing support for resilience and coping strategies is a crucial approach to addressing this ongoing issue.

This study confirmed Hypothesis 1, which proposed that resilience serves as a key mediator between nurse stress and professional commitment. Resilience, as a form of psychological flexibility, enables individuals to adapt to and recover from adversity and stress. Previous research has indicated that higher resilience levels are associated with reduced emotional exhaustion and stress, as well as increased work engagement and job satisfaction [27]. Furthermore, resilience training has been shown to enhance nurses' adaptability, reducing anxiety, depression, and burnout [28]. Specific resilience-building strategies include: mindfulness and relaxation training, psychoeducation, emotion regulation techniques, cognitive strategies, problem-solving skills,

strengthening internal and external support resources. These strategies have demonstrated moderate positive effects in previous studies [29]. However, resilience in nursing is a self-regulatory process that involves both internal factors (e.g., cognitive appraisal and adaptive thinking) and external resources (e.g., workplace support and clinical administrative oversight) [30]. When organizational support is insufficient, nurses' self-regulatory capacity becomes constrained, potentially leading to negative consequences or an imbalance between stress and professional commitment. Therefore, governments and healthcare institutions should implement supportive policies, such as: optimizing nurse-to-patient ratios, effective workload management, enhancing workplace support systems, providing continuous education and professional development. These measures will help nurses leverage resilience to maintain professional commitment in high-stress environments.

This study confirmed Hypothesis 2, which proposed that coping serves as a key mediator between nurse stress and professional commitment. Coping refers to an individual's cognitive and behavioral processes used to evaluate events -including external and internal demands, challenges, and conflicts -while seeking internal and external support and resources to effectively manage stress [8]. This process is dynamic and continuously adjusts based on situational changes and individual psychological states. Recent research on COVID-19-related stress has indicated that nurses can adapt to workplace stress by employing strategies such as acceptance, religious beliefs, planning, and positive reframing, while integrating ethical and mindfulness-based approaches to enhance infection prevention training and professional competency [31,32]. Additionally, psychosocial coping strategies, communication with family members, and helping others are considered flexible

coping mechanisms commonly used by nurses. Studies have shown that nurses with stronger coping abilities experience lower levels of stress [33]. However, some research has revealed that certain nurses exhibited maladaptive coping tendencies during the pandemic, such as fearing the risk of infecting family members or regretting their career choice [32]. When nurses face negative coping responses, psychological intervention techniques may offer effective support, including: cognitive processing therapy, emotional freedom techniques, prolonged exposure therapy, eye movement desensitization and reprocessing. These techniques have been shown to reduce psychological burdens associated with pandemic-related stress, assist nurses in developing adaptive coping strategies, and enable them to gradually reintegrate into society and return to their professional nursing roles through continuous adaptation and accumulated experience [34]. Therefore, to ensure nurses' psychological well-being, healthcare institutions and related support systems should provide necessary assistance and resources to enhance coping abilities, ultimately improving job stability and strengthening professional commitment.

The findings of this study confirmed Hypothesis 3, demonstrating that the relationship between nurse stress and professional commitment is mediated through the influence of resilience on coping strategies. The results indicate that higher levels of nurse stress significantly reduce professional commitment, while resilience and coping strategies play crucial mediating roles in mitigating this negative impact. As a core indicator of psychological resilience, resilience helps nurses maintain emotional stability and professional engagement in high-stress environments. Additionally, appropriate coping strategies - particularly problem-focused coping - enable nurses to effectively

manage work challenges, thereby reducing the adverse effects of stress on professional commitment. Despite the gradual decline of the COVID-19 pandemic, the nursing profession continues to face workforce shortages and increasing vacancy rates. This trend suggests that beyond demographic challenges such as low birth rates, the nursing work environment requires more proactive policies and resource support. By enhancing professional knowledge and cultivating a rational yet optimistic mindset, nurses can improve their ability to manage stress, thereby reinforcing professional identity and fostering greater enthusiasm for their work. Overall, strengthening nurses' psychological well-being, professional commitment, and stress management skills will contribute to greater long-term retention, stabilizing the nursing workforce, and ultimately ensuring high-quality healthcare services.

Limitations

This cross-sectional design precludes causal inference. Data were drawn from one medical center in central Taiwan, limiting generalizability. Future longitudinal and multi-center studies are recommended to verify causal pathways and broaden applicability.

Conclusion

Nurse stress adversely affects professional commitment, while resilience and coping serve as crucial mediators that buffer this relationship. Enhancing these psychological resources through organizational and educational interventions can mitigate stress, improve commitment, and ensure the sustainability of nursing care quality.

Figure 1. Hypothesized serial mediation model linking nurse stress and nurse professional commitment through resilience and coping as serial mediators

Legend: Nurse stress was negatively associated with resilience (a^1 = -0.1131, $p < .001$) and coping (a^2= -0.0198, $p = .0011$). Resilience was positively associated with coping (a^3 = 0.1525, $p < .001$). The direct effects of mediating variables on nurse professional commitment showed that the effects of resilience (b^1 = 0.2439, $p < .001$) and coping (b^2 = 0.4045, $p < .001$) were significant. The direct effect of nurse stress on nurse professional commitment was also significant (c′ = -0.0382, $p = .0011$). The total effect (c = -0.0808, $p < .001$) was significantly negatively correlated.

References

1. González-Gil MT, González-Blázquez C, Parro-Moreno AI, Pedraz-Marcos A, Palmar-Santos A, Otero-García L, et al. Nurses' perceptions and demands regarding COVID-19 care delivery in critical care units and hospital emergency services. Intensive Crit Care Nurs. 2021;62:102966. https://doi.org/10.1016/j.iccn.2020.102966

2. Quesada-Puga C, Izquierdo-Espin FJ, Membrive-Jiménez MJ, Aguayo-Estremera R, Cañadas-De La Fuente GA, et al. Job satisfaction and burnout syndrome among intensive-care unit nurses: A systematic review and meta-analysis. Intensive Crit Care Nurs. 2024;82:103660. https://doi.org/10.1016/j.iccn.2024.103660

3. Meyer JP, Allen NJ, Smith CA. Commitment to organizations and occupations: Extension and

test of a three-component conceptualization. J Appl Psychol. 1993;78(4):538.

4. Nabi Foodani M, Abbasi Dolatabadi Z, Goudarzian AH, Mosazadeh H, Poortaghi S. Factors Effective in Lowering Professional Commitment in Nursing and Strategies to Address Them: A Scoping Review. SAGE Open Nurs. 2024;10:23779608241299291. https://doi.org/10.1177/23779608241299291

5. Grotberg EH. The International Resilience Project: Research and Application. 1995.

6. Hart PL, Brannan JD, De Chesnay M. Resilience in nurses: an integrative review. J Nurs Manag. 2014;22(6):720-734. https://doi.org/10.1111/j.1365-2834.2012.01485.x

7. Castillo-González A, Velando-Soriano A, De La Fuente-Solana EI, Martos-Cabrera BM, Membrive-Jiménez MJ, Lucía RB, et al. Relation and effect of resilience on burnout in nurses: A literature review and meta-analysis. Int Nurs Rev. 2024;71(1):160-167. https://doi.org/10.1111/inr.12838

8. Lazarus RS, Folkman S. Stress, appraisal, and coping: New York: Springer Publishing Company; 1984.

9. Betke K, Basińska MA, Andruszkiewicz A. Sense of coherence and strategies for coping with stress among nurses. BMC Nurs. 2021;20(1):107. https://doi.org/10.1186/s12912-021-00631-1

10. Maresca G, Corallo F, Catanese G, Formica C, Lo Buono V. Coping Strategies of Healthcare Professionals with Burnout Syndrome: A Systematic Review. Medicina (Kaunas). 2022;58(2):327. https://doi.org/10.3390/medicina58020327

11. Croghan IT, Chesak SS, Adusumalli J, Fischer KM, Beck EW, Patel SR, et al. Stress,

Resilience, and Coping of Healthcare Workers during the COVID-19 Pandemic. J Prim Care Community Health. 2021;12:21501327211008448. https://doi.org/10.1177/21501327211008448

12. Labrague LJ. Psychological resilience, coping behaviours and social support among health care workers during the COVID-19 pandemic: A systematic review of quantitative studies. J Nurs Manag. 2021;29(7):1893-1905. https://doi.org/10.1111/jonm.13336

13. Zhang X, Xie J, Wu W, Cao L, Jiang Z, Li Z, Li Y. The mediation effect of mental resilience between stress and coping style among parents of children with cochlear implants: Cross-sectional study. J Pediatr Nurs. 2024;74:1-9. https://doi.org/10.1016/j.pedn.2023.10.042

14. Yu X, Yang Y, He B. The effect of athletes' training satisfaction on competitive state anxiety-a chain-mediated effect based on psychological resilience and coping strategies. Front Psychol. 2024;15:1409757. https://doi.org/10.3389/fpsyg.2024.1409757

15. Zhang Y, Ding X, Liu Y, Han Y, Wang G, Cai M, et al. The Relationship Between Social Support and Suicide Resilience in Chinese Cancer Patients: A Serial Multiple Mediation Model Through Self-care Self-efficacy and Meaning in Life. Cancer nursing. 2024;47(4): E236–E244. https://doi.org/10.1097/NCC.0000000000001202

16. Faul F, Erdfelder E, Buchner A, Lang AG. Statistical power analyses using G*Power 3.1: tests for correlation and regression analyses. Behav Res Methods. 2009;41(4):1149-60. https://doi.org/10.3758/BRM.41.4.1149

17. Benoliel JQ, McCorkle R, Georgiadou F, Denton T, Spitzer A. Measurement of stress in clinical nursing. Cancer Nurs. 1990;13(4):221-218. https://doi.org/10.1097/00002820-199008000-

00003

18. Tsai S-L, Chen M-L. A test of the reliability and validity of Nurse Stress Checklist. J Nurs Res. 1996;4(4):355-362.

19. Wagnild GM, Young HM. Development and psychometric evaluation of the Resilience Scale. J Nurs Meas. 1993;1(2):165-178.

20. Carver CS. You want to measure coping but your protocol's too long: consider the brief COPE. Int J Behav Med. 1997;4(1):92-100. https://doi.org/10.1207/s15327558ijbm0401_6

21. Lin C-J, Wang H-C, Li T-C, Huang L-C. Reliability and Validity of Nurses' Job Satisfaction Scale and Nurses' Professional Commitment. Mid-Taiwan Journal of Medicine. 2007;12(2): 65-75.

22. Hayes AF. Mediation, moderation, and conditional process analysis. Introduction to mediation, moderation, and conditional process analysis: A regression-based approach. 2013;1(6):12-20.

23. Johnston MG, Faulkner C. A bootstrap approach is a superior statistical method for the comparison of non-normal data with differing variances. New Phytol. 2021;230(1):23-26. https://doi.org/10.1111/nph.17159

24. Liao H, Liang R, He H, Huang Y, Liu M. Work stress, burnout, occupational commitment, and social support among Chinese pediatric nurses: A moderated mediation model. J Pediatr Nurs. 2022;67:e16-e23. https://doi.org/10.1016/j.pedn.2022.10.009

25. Ministry of Health and Welfare. Ministry of Health and Welfare 2023 Hospital Nursing Service Volume Survey Result 2023 [Available from: https://nurse.mohw.gov.tw/cp-155-2079-e1359-2.html.]

26. Sundram BM, Vallasamy SK, Muhadi U. Exploring the Multifaceted Nature of Occupational Stress Among Nurses: A Systematic Review. Kesmas. 2024;19(2):121-128.

27. Sharma S, Talib P, Singh G. Review of studies on stress, job satisfaction and resilience among nursing professionals. Indian Journal of Continuing Nursing Education. 2021;22(2):215-220.

28. Zhai X, Ren LN, Liu Y, Liu CJ, Su XG, Feng BE. Resilience Training for Nurses: A Meta-analysis. J Hosp Palliat Nurs. 2021;23(6):544-550. https://doi.org/10.1097/njh.0000000000000791

29. Kunzler AM, Chmitorz A, Röthke N, Staginnus M, Schäfer SK, Stoffers-Winterling J, et al. Interventions to foster resilience in nursing staff: A systematic review and meta-analyses of pre-pandemic evidence. Int J Nurs Stud. 2022;134:104312. https://doi.org/10.1016/j.ijnurstu.2022.104312

30. McLarnon MJ, Rothstein MG. Development and initial validation of the workplace resilience inventory. Journal of Personnel Psychology. 2013;12(2):63-73.

31. Sierakowska M, Doroszkiewicz H. Stress coping strategies used by nurses during the COVID-19 pandemic. PeerJ. 2022;10:e13288. https://doi.org/10.7717/peerj.13288

32. Engelbrecht MC, Heunis JC, Kigozi NG. Post-Traumatic Stress and Coping Strategies of South African Nurses during the Second Wave of the COVID-19 Pandemic. Int J Environ Res Public Health. 2021;18(15):7919. https://doi.org/10.3390/ijerph18157919

33. Magdi HM. Stress and resilient coping among nurses: Lessons learned from the COVID-19 pandemic. Psych. 2022;4(3):615-625.

34. Riedel B, Horen SR, Reynolds A, Hamidian Jahromi A. Mental Health Disorders in Nurses

During the COVID-19 Pandemic: Implications and Coping Strategies. Front Public Health. 2021;9:707358. https://doi.org/10.3389/fpubh.2021.707358

Figure

Click here to access/download;Figure;Figure 1.jpg

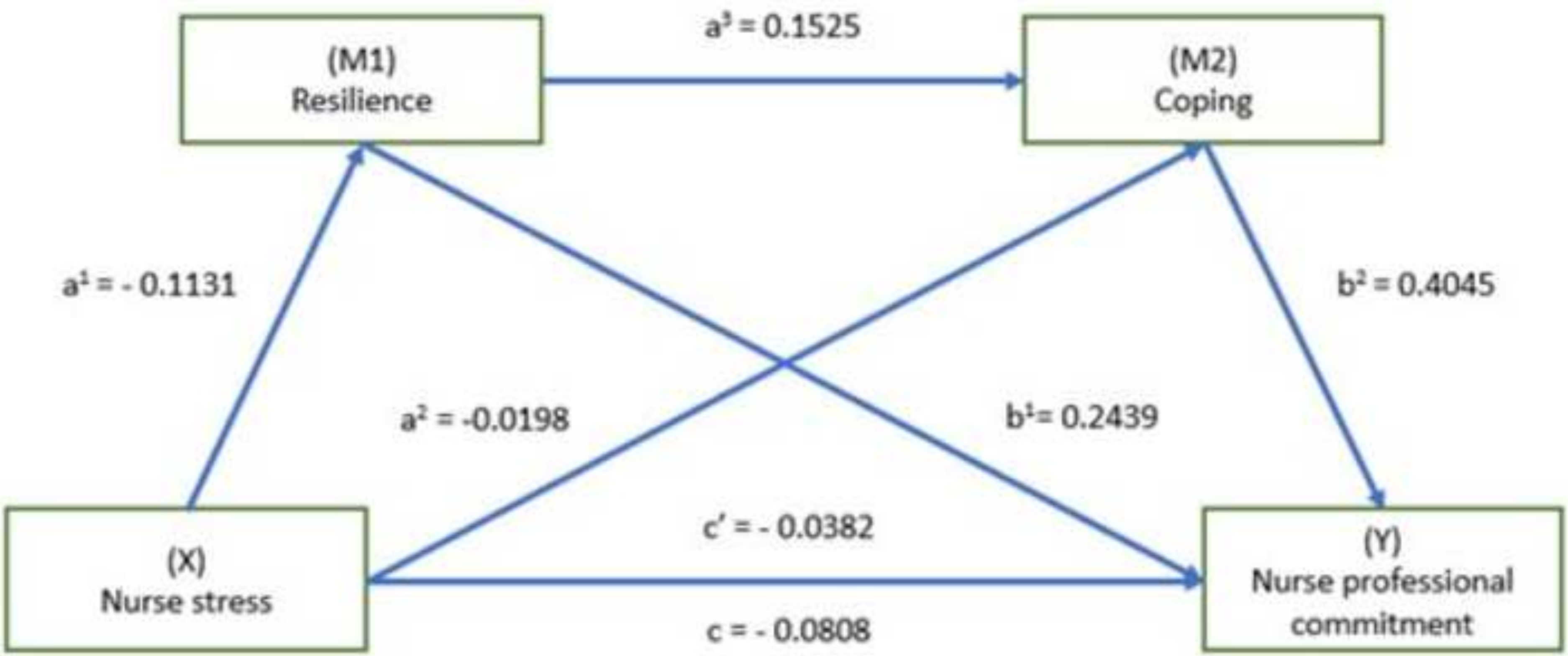

Table 1. Differences in Nurse stress by general characteristics (n = 345)

Variables	N=345	Range / %	Nurse stress (Mean ± SD)	p
Age (year)	32.00±8.62	22.0-60.0		0.953
20-29	186	53.9%	142.88 ± 56.76	
30-39	84	24.3%	140.36 ± 53.92	
40-49	59	17.1%	145.29 ± 58.80	
≥50	16	4.6%	139.06 ± 51.28	
Job tenure - Nursing experience (years)	9.00±8.21	1.0-38.0		0.790
1-<3	88	25.5%	140.24 ± 64.94	
3-<6	75	21.7%	144.73 ± 49.03	
6-<9	50	14.5%	149.26 ± 58.61	
9-<12	40	11.6%	134.97 ± 52.89	
≥12	92	26.7%	142.45 ± 52.50	
Gender				0.435
Female	318	92.2 %	143.19 ± 55.95	
Male	27	7.8 %	134.41 ± 56.89	
Marital Relationship				0.956
Single	257	74.5 %	141.99 ± 55.70	
Married	86	24.9 %	143.93 ± 56.37	
Divorce	2	0.6 %	147.00 ± 117.38	
Education level				0.367
College	20	5.8 %	126.15 ± 45.42	
University	301	87.2 %	143.11 ± 55.94	
Master's and Doctoral	24	7 %	148.42 ± 63.95	
Affiliated units				<0.001
Medical & surgical ward	183	53.0 %	153.80 ± 57.94	
Obstetric & pediatric ward	53	15.4 %	124.13 ± 42.81	
Emergency & critical care unites	109	31.6 %	132.47 ± 54.41	

Table 2: Pearson correlations test between nurse stress, resilience, coping, and nurse professional commitment

Variables	Mean (SD)	1	2	3	4
1. Nurse stress	142.50±55.99	1			
2. Resilience	118.39±21.76	-0.291*	1		
3. Coping	77.96±6.96	-0.296*	0.523*	1	
4. Nurse professional commitment	67.46±13.90	-0.338*	0.536*	0.451*	1

*$p < .01$ (2-tailed).

Table 3 : Bootstrapping Indirect Effects and 95% CIs for the Final Mediational Model

	Point Estimate	Boot SE	Boot LLCI	Boot ULCI
Effect				
A total indirect effect of X on Y	-0.0426	0.0078	-0.0585	-0.0277
Indirect effect 1: X[a] → M1[b] → Y[c]	-0.0276	0.0072	-0.0429	-0.0146
Indirect effect 2: X → M2[b] → Y	-0.0080	0.0032	-0.0150	-0.0025
Indirect effect 3: X → M1 → M2 → Y	-0.0070	0.0025	-0.0124	-0.0027
Contrasts				
Model 1[e] vs model 2[f]	-0.0196	0.0090	-0.0378	-0.0023
Model 1 vs model 3[g]	-0.0206	0.0072	-0.0364	-0.0076
Model 2 vs model 3	-0.0010	0.0036	-0.0082	0.0060

Abbreviations: CI, confidence interval; LL, lower level; UL, upper level.

[a]X = Nurse stress

[b]M1 = Resilience

[c]Y = Nurse professional commitment

dM2 = Coping

[e]Model 1 = Nurse Stress → Resilience → Nurse professional commitment.

[f]Model 2 = Nurse Stress → Coping → Nurse professional commitment.

[g]Model 3 = Nurse Stress → Resilience → Coping → Nurse professional commitment.
