# Supplementary material for: The relationship between nurse stress and professional commitment in clinical nurses: A serial multiple mediation model through resilience and coping
Source: PLoS One. 2026 Aug 24;21(8):e0356880. doi: 10.1371/journal.pone.0356880 (PMC13502600; doi:10.1371/journal.pone.0356880)
Supplement: Supplementary file 2 [file pone.0356880.s003.docx]

**Dear Academic Editor and Reviewer's**

Response to Editorial Requests Regarding Data Availability and Ethics Statement

Thank you for your comments regarding data availability and ethics requirements. We have addressed the issues as follows:

Response to Points 3 and 4: Data Availability and Data Access Contact Information

The dataset used in this study contains sensitive human participant information collected under Institutional Review Board (IRB) approval. Although all direct identifiers have been removed, the data include demographic and occupational variables that may potentially allow participant re-identification when combined with other information. Therefore, public deposition of the dataset is restricted to protect participant confidentiality and privacy, in accordance with the requirements of the Institutional Review Board of Chung Shan Medical University Hospital.

The restrictions on data sharing were imposed by: Institutional Review Board (IRB), Chung Shan Medical University Hospital Approval Number: CS2-23171

Interested researchers who meet the criteria for access to confidential data may submit requests to the following non-author institutional contact: Institutional Review Board (IRB) Chung Shan Medical University Hospital. Contact Person: Pin-Hsun Lin, E-mail: cshe1625@csh.org.tw

For scientific inquiries regarding the study, researchers may additionally contact the corresponding author: Chia-En Hsieh , E-mail: chiaen69918@gmail.com

The original research data are securely stored at Chung Shan Medical University Hospital in accordance with institutional research data management regulations. Data will be maintained in secure institutional storage systems and retained according to the hospital's research governance and data retention policies, ensuring long-term availability for qualified researchers upon reasonable request and with IRB approval.

Accordingly, the Data Availability Statement has been revised as follows:

Data Availability Statement : The data underlying the results presented in this study contain sensitive participant information and cannot be publicly shared due to ethical and legal restrictions imposed by the Institutional Review Board of Chung Shan Medical University Hospital (Approval No. CS2-23171). Qualified researchers may request access to the data by contacting the Institutional Review Board of Chung Shan Medical University Hospital (Contact Person: Pin-Hsun Lin; E-mail: cshe1625@csh.org.tw). Data access will be provided to researchers who meet the criteria for access to confidential data and obtain appropriate ethical approval where required.

Response to Point 5: Ethics Statement

We have reviewed the manuscript and confirmed that the Ethics Statement appears only in the Methods section. Any duplicate ethics-related statements appearing elsewhere in the manuscript have been removed. The Ethics Statement remains included in the Methods section to ensure that it will be published with the article.

**All revisions made in response to the reviewers' and editor's comments are highlighted in yellow in the revised manuscript. The highlighted text indicates the sections that have been modified, added, or clarified.**

**Response to Reviewer #1**

We sincerely thank the reviewer for the constructive and insightful comments. We have carefully revised the manuscript to improve clarity, methodological transparency, internal consistency, and the appropriateness of interpretation. All changes have been clearly indicated in the revised manuscript. Our detailed responses are provided below.

Comment 1: Clarity and consistency (sample & setting)

Reviewer comment: Some aspects related to the sample and setting are not described in a fully consistent manner.

**Response:** Thank you for this important comment. We have carefully reviewed and revised all relevant sections (Abstract, Methods, Results) to ensure consistency in describing the study setting, departments, and sampling framework.

**Revised content :**

1. This cross-sectional study was conducted at a single medical center and involved 345 clinical nurses recruited from internal medical and surgical wards, obstetric and pediatric wards, and emergency and critical care units. The valid response rate was 97.2%. All participants were recruited from inpatient units within the same medical center, ensuring a consistent clinical setting across all departments.

**Location revised:** Abstract (Materials and Methods)

2. The participants were nurses from the internal medical and surgical wards, obstetric and pediatric wards, and emergency and critical care units.

A total of 355 eligible nurses were invited to participate, of whom 345 provided complete questionnaires suitable for analysis, yielding a valid response rate of 97.2%

**Location revised**: Methods (Design and Participants)

3. Participants were recruited from medical and surgical wards (n = 183, 53.0%), obstetric and pediatric wards (n = 53, 15.4%), and emergency and critical care units (n = 109, 31.6%)

**Location revised:** Results (Participant characteristics)

Comment 2: Methodological transparency & analysis clarity

Reviewer comment: Analytical strategy requires clearer and more transparent reporting.

**Response:** We have expanded the Statistical Analysis section to clearly describe: variable roles, covariates, rationale for PROCESS Model 6, and bootstrap justification.

**Revised text:** Statistical analyses:

To enhance transparency, nurse stress was specified as an independent variable, professional commitment as the dependent variable, and resilience and coping as serial mediators in the model.

Serial mediation analysis was conducted using PROCESS macro Model 6, which allows the testing of both independent and sequential indirect effects. This model was selected based on a theoretical framework derived from stress and coping theory. A bootstrap resampling procedure with 2,000 iterations was applied to estimate indirect effects and their 95% confidence intervals because this method does not assume normality and provides more a robust inference.

Bootstrap resampling of 2,000 iterations was used to test the indirect effects of one independent variable (nurse stress), two mediating variables (resilience and coping), and one dependent variable (nurse professional commitment). Demographic characteristics related to professional commitment were included as covariates in the analysis. The significance level was set at .05 (two-tailed test).

**Location revised:** Statistical analyses.

Comment 3: Overinterpretation / causal language

Reviewer comment: Language implies causality despite cross-sectional design.

**Response:** We fully agree.

**Revised text:**

1. Discussion

This study examined the relationship between nurse stress and professional commitment, as well as the mediating roles of resilience and coping among clinical nurses. The findings demonstrated that nurse stress was significantly and negatively associated with professional commitment, both directly and indirectly through the mediating effects of resilience and coping strategies. These findings should be interpreted as associative rather than causal because of the cross-sectional design. Additionally, the results confirmed that resilience influences coping, further contributing to the serial-multiple mediation effect in this relationship.

**Revised section:** Discussion section, Paragraph 1.

2. Limitations

Due to the cross-sectional design, causal relationships cannot be established, and the findings should be interpreted as associations rather than causal effects.

**Revised section:** Limitations

Comment 4: Conclusion too brief & generic

Reviewer comment: Conclusion should better summarize findings and be proportionate.

**Response:** We have substantially expanded and refined the conclusion to: clearly summarize main findings, avoid overstatement, align with study design.

**Revised text:** Conclusion

Nurse stress was significantly associated with lower professional commitment among clinical nurses, with resilience and coping functioning as important mediating factors. These findings highlight the potential importance of psychological resources in understanding how nurses respond to workplace stress. However, given the cross-sectional design and single-center setting, the results should be interpreted with caution as associative rather than causal. Future longitudinal and multi-center studies are warranted to further validate these relationships and explore causal the pathways. Interventions aimed at enhancing resilience and adaptive coping may be beneficial for supporting nurses’ professional commitment.

**Location revised:** Conclusion.

**Reviewer 2 has contacted us to clarify that the comments they provided were submitted in error.**

No response to those comments is expected.

**Response to Reviewer #3**

We sincerely thank Reviewer #3 for the insightful and constructive comments. We greatly appreciate the reviewer’s recognition of the importance of our study topic and the analytical contribution of the serial multiple mediation model. We have carefully revised the manuscript in response to all comments and suggestions. Detailed responses are provided below.

Comment 1: The Brief-COPE aggregation into a single global coping score is methodologically debatable. The authors should consider complementary analyses using adaptive and maladaptive coping subscales.

**Response:** Thank you for this important methodological suggestion. We acknowledge that the Brief-COPE consists of multiple theoretically distinct coping strategies. In the present study, we adopted a global coping score to examine the overall mediating effect of coping within the serial mediation framework and to maintain model parsimony. However, we agree that combining adaptive and maladaptive coping strategies may obscure their differential effects. Accordingly, we have added this issue to the limitations section and discussed the need for future studies to further classify coping strategies into adaptive and maladaptive dimensions to better clarify their distinct mediating roles and implications for nursing management interventions.

**Revised section:**

The present study utilized a global coping score derived from the Brief-COPE. As coping strategies may include both adaptive and maladaptive dimensions, future studies are encouraged to separately examine these coping subtypes to better clarify their differential mediating effects. Future longitudinal and multi-center studies are recommended to verify causal pathways and broaden their applicability.

**Revised section:** Limitations section.

Comment 2: The discussion regarding convenience sampling and recruitment from a single medical center should be expanded to address generalizability.

**Response:** Thank you for this valuable comment. The present study was conducted in a large medical center. Compared with smaller community hospitals, large medical centers may differ in several aspects, including patient acuity, workload intensity, staffing patterns, organizational resources, and workplace support systems. However, due to the study setting and sampling conditions, these institutional differences could not be directly compared or systematically evaluated in the current study. Therefore, this issue has been acknowledged and discussed in the limitations section.

**Revised section:**

participants were recruited using convenience sampling from a single large medical center in central Taiwan, which may have limited the generalizability of our findings. Compared with smaller community hospitals, large medical centers may differ in patient acuity, workload intensity, staffing patterns, organizational resources, and workplace support systems.

**Revised section:** Limitations section.

Comment 3: The manuscript should include a formal Data Availability Statement.

**Response:** Thank you for the suggestion. A formal Data Availability Statement has now been added to the manuscript.

**Added text:**

Data Availability Statement :

Ethical restrictions prohibit the authors from making the data publicly available to protect the confidentiality and privacy of patients. Interested researchers can submit data access requests to the Chung Shan Medical University Hospital Institutional.

Data Access / Ethics Committee:

Institutional Review Board of the Chung Shan Medical University Hospital of CS2-23171; Contact Person: Pin-Hsun Lin; E-mail: cshe1625@csh.org.tw. Additionally, interested researchers may contact the corresponding author, Chia-En Hsieh (chiaen69918@gmail.com)

**Revised section:** Added a “Data Availability Statement” before the References section.

Comment 4: The authors should explain why PROCESS Model 6 was selected instead of SEM approaches.

**Response:** Thank you for this important methodological comment. We have added an explanation in the Statistical Analyses section clarifying that PROCESS Model 6 was selected because the study aimed to test directional serial indirect effects among observed variables using bootstrap estimation. In addition, the sample size and study design were considered appropriate for regression-based mediation analysis. We also acknowledged that SEM approaches may provide additional advantages for latent variable modeling and model fit evaluation in future studies.

**Added text:**

PROCESS Model 6 was selected because the study aimed to examine the sequential indirect effects among observed variables using a regression-based bootstrap mediation approach. Compared with SEM, PROCESS is an efficient and widely accepted method for testing serial mediation effects in cross-sectional studies with observed variables and moderate sample sizes.

**Revised section:** Statistical Analyses section.

Comment 5: The discussion should further explore the unexplained direct effect and possible organizational-level factors influencing professional commitment.

**Response:** Thank you for this insightful suggestion. We agree that the persistence of a significant direct effect indicates that additional factors beyond resilience and coping may contribute to professional commitment. Therefore, we expanded the discussion to include organizational-level influences such as workload burden, staffing adequacy, leadership support, workplace culture, scheduling flexibility, and institutional support systems.

**Revised section:**

Although resilience and coping partially mediated the relationship between nurse stress and professional commitment, the direct effect of stress remained significant, suggesting that additional organizational factors may also influence professional commitment. Factors such as staffing adequacy, workload intensity, leadership support, workplace culture, scheduling flexibility, and institutional support systems may substantially affect nurses’ professional engagement and retention intentions. Therefore, future studies should incorporate these organizational variables to develop a more comprehensive explanatory framework regarding the factors influencing nurses’ professional commitment.

**Revised section:** Discussion section, Paragraph 6.

Comment 6: The discussion should better balance individual-level interventions with organizational and system-level changes.

Comment 7: Please ensure references are updated with recent literature (2024–2026).

**Response:** Thank you for this important recommendation. We have strengthened the discussion by emphasizing that professional commitment is influenced not only by individual psychological resources but also by organizational and systemic workplace conditions. Additional discussion regarding safe staffing, workload management, workplace support systems, and institutional policies has been incorporated. We carefully reviewed and updated the reference list by incorporating additional recent literature related to nurse retention, resilience, coping, stress, and mental health in post-pandemic nursing contexts.

**Revised section:**

Despite facing considerable workplace challenges, nurses continued to demonstrate strong professional commitment and dedication to patient care, particularly as frontline healthcare providers and first responders. The findings indicated that respondents experienced moderate to high levels of perceived work stress, accompanied by relatively low psychological resilience, while maintaining moderate levels of work engagement and self-efficacy [35]. Despite these stressors, many nurses actively developed personal coping strategies and adaptive mechanisms to manage occupational demands [36]. Effective stress adjustment strategies, including coping abilities and psychological hardiness, appeared to strengthen nurses’ commitment to their professional practice and support their continued engagement in clinical responsibilities [37]. These findings highlight the importance of enhancing resilience-building and stress-management interventions to sustain nurses’ professional commitment and overall well-being in demanding healthcare environments. Although resilience and coping partially mediated the relationship between nurse stress and professional commitment, the direct effect of stress remained significant, suggesting that additional organizational factors may also influence professional commitment. Factors such as staffing adequacy, workload intensity, leadership support, workplace culture, scheduling flexibility, and institutional support systems may substantially affect nurses’ professional engagement and retention intentions. Therefore, future studies should incorporate these organizational variables to develop a more comprehensive explanatory framework regarding the factors influencing nurses’ professional commitment.

**Revised section:** Discussion section, Paragraphs 6.

**References section:**

35. Bartoszuk J, Sierakowska M. Selected aspects of the psychosocial functioning of medical personnel of emergency medical teams-a cross-sectional study. Front Public Health. 2026;13:1754424. https://doi.org/10.3389/fpubh.2025.1754424

36. Song L, Du L, Xu L, Wu M, Li X. Navigating Challenges and Building Resilience: Qualitative Insights into Disaster Nursing Preparedness in Chinese Non-First-Tier Cities. Disaster Med Public Health Prep. 2025;19:e250. https://doi.org/10.1017/dmp.2025.10177

37. Druart MC, Blanchard PY. Personal resources used by nurses enrolled in an advanced practice curriculum during the COVID-19 pandemic in France: Sequential mixed research. Rech Soins Infirm. 2022;148(1):89-106. https://doi.org/10.3917/rsi.148.0089

Comment 8: Please verify that Figure 1 explicitly labels coefficients and significance levels consistently.

**Response:** Thank you for the careful observation. We reviewed and revised Figure 1 and its legend to ensure that all path coefficients and corresponding significance levels are clearly labeled and consistently reported throughout the manuscript text.

**Revised section:**

Legend: Nurse stress was negatively associated with resilience (a^1^ = -0.1131, p < 0.001) and coping (a^2^= -0.0198, p = 0.001). Resilience was positively associated with coping (a^3^ = 0.1525, p < 0.001). The direct effects of mediating variables on nurse professional commitment showed that the effects of resilience (b^1^ = 0.2439, p < 0.001) and coping (b^2^ = 0.4045, p < 0.001) were significant. The direct effect of nurse stress on nurse professional commitment was also significant (c′ = -0.0382, p = 0.001). The total effect (c = -0.0808, p < 0.001) was significantly negatively correlated.

**Revised section:** Figure 1 and its legend.

**Response to Reviewer #4**

Thank you very much for your valuable comments and constructive suggestions. We sincerely appreciate your careful review of our manuscript. In response to your comments, we have revised the manuscript accordingly. The detailed responses are provided below.

Comment 1: Minor grammatical errors need correcting.

**Response:** Thank you for your suggestion. We carefully reviewed the entire manuscript and corrected minor grammatical, punctuation, and sentence structure issues to improve clarity, readability, and academic writing quality throughout the manuscript.

**Revisions made:** Corrected grammatical inconsistencies and sentence structures throughout the manuscript. Improved wording precision and readability in the Introduction, Methods, Discussion, and Conclusion sections. Revised several long or unclear sentences for better academic expression.

Comment 2: Regarding the Limitations section - please provide greater clarity and examples of limitations and study results do not imply causal relationships.

**Response:** Thank you for this important suggestion. We revised the Limitations section to provide clearer explanations regarding the study limitations and explicitly clarified that the findings do not imply causal relationships due to the cross-sectional design. We also added additional examples regarding sampling limitations, self-reported measurements, and potential response bias.

**Revised text:**

Due to the cross-sectional design, causal relationships cannot be established, and the findings should be interpreted as associations rather than causal effects. In addition, participants were recruited using convenience sampling from a single large medical center in central Taiwan, which may have limited the generalizability of our findings. Compared with smaller community hospitals, large medical centers may differ in patient acuity, workload intensity, staffing patterns, organizational resources, and workplace support systems. These institutional characteristics may influence nurses’ stress experiences, coping processes, resilience, and professional commitment. All study variables were assessed using self-reported questionnaires, which may introduce recall bias or social desirability bias. Furthermore, the present study utilized a global coping score derived from the Brief-COPE. As coping strategies may include both adaptive and maladaptive dimensions, future studies are encouraged to separately examine these coping subtypes to better clarify their differential mediating effects. Future longitudinal and multi-center studies are recommended to verify causal pathways and broaden their applicability.

**Revised section:** Limitations section.

Comment 3: Consider restructuring sentences regarding confirmation of hypotheses 1, 2, and 3 to introduce hypotheses again prior to confirmation from the data.

**Response:** Thank you for this valuable recommendation. We revised the Discussion section by restructuring the paragraphs related to Hypotheses 1, 2, and 3. Specifically, we now reintroduce each hypothesis before discussing the corresponding findings and interpretations, which improves logical flow and readability.

**Revised text:**

1. Hypothesis 1 proposed that resilience mediates the relationship between nurse stress and professional commitment. The findings of this study supported this hypothesis, indicating that resilience serves as a key psychological mediator between nurse stress and professional commitment.

**Revised section:** Discussion section, Paragraph 3.

2. Hypothesis 2 proposed that coping would mediate the relationship between nurse stress and professional commitment. The findings supported this hypothesis, demonstrating that coping functions as an important mediator between nurse stress and professional commitment.

**Revised section**: Discussion section, Paragraph 4.

3. Hypothesis 3 proposed that resilience and coping operate as serial mediators in the relationship between nurse stress and professional commitment. These findings supported our hypothesis and demonstrated a significant serial mediation pathway through resilience and coping.

**Revised section:** Discussion section, Paragraph 5

We sincerely appreciate the reviewer’s insightful comments, which have helped improve the clarity and overall quality of the manuscript.

Sincerely,

Chia-En Hsieh, RN, NP, Assistant Professor, PhD.

Liver transplantation center, Chung Shan Medical University Hospital, Taichung, Taiwan

e-Mail : chiaen69918@gmail.com
