# Supplementary material for: The relationship between nurse stress and professional commitment in clinical nurses: A serial multiple mediation model through resilience and coping
Source: PLoS One. 2026 Aug 24;21(8):e0356880. doi: 10.1371/journal.pone.0356880 (PMC13502600; doi:10.1371/journal.pone.0356880)
Supplement: Supplementary file 3 [file pone.0356880.s004.docx]

**Dear Academic Editor and Reviewer's**

**All revisions made in response to the reviewers' and editor's comments are highlighted in yellow in the revised manuscript. The highlighted text indicates the sections that have been modified, added, or clarified.**

**Response to Reviewer #1**

Thank you very much for your positive evaluation of our revised manuscript and for recognizing the improvements in the clarity of the study design, sample description, mediation analysis, and acknowledgment of the cross-sectional nature of the study.

We sincerely appreciate your constructive suggestions. We have carefully revised the manuscript accordingly. Detailed responses to each comment are provided below.

**Comment 1: Please further reduce causal language throughout the manuscript, especially in the Discussion and Conclusion. Terms such as “impact,” “influences,” and “effects” should be replaced, where appropriate, with more cautious wording such as “association” or “is associated with.”**

**Response:** Thank you for this important suggestion. We carefully reviewed the entire manuscript and revised expressions implying causality to more appropriately reflect the cross-sectional nature of the study. Terms such as “impact,” “influences,” “effects,” and “reduce” were replaced with more cautious expressions including “association,” “is associated with,” “may contribute to,” and “may be beneficial.” Revisions were made throughout the Introduction, Discussion, Conclusion, and Figure Legend.

**Location revised:** Introduction section, Paragraph 6.

Discussion section, Paragraph 5.

Conclusion section.

Figure Legend.

**Comment 2: Please clarify the rationale for using a global Brief-COPE score, since this instrument includes both adaptive and maladaptive coping strategies. If no further analyses are performed, this limitation should be emphasized more clearly.**

**Response:** Thank you for this insightful comment. The Brief-COPE total score was used because our primary objective was to examine the overall coping tendency within the proposed serial mediation framework rather than individual coping subtypes. We have clarified this rationale in the Measures section and further strengthened the Limitations section by emphasizing that the global coping score may obscure differential effects between adaptive and maladaptive coping strategies. We also recommend that future studies separately investigate Brief-COPE subdimensions.

**Added text:** In the present study, an overall Brief-COPE score was used to represent participants' general coping tendencies within the proposed mediation framework. This approach was selected to capture the overall utilization of coping responses rather than specific coping subtypes. However, because the Brief-COPE encompasses both adaptive and maladaptive coping strategies, interpretation of the global score should be made cautiously.

**Location revised:** Methods & Measures section

**Revised content:** Furthermore, all study variables were assessed using self-reported questionnaires, which may introduce recall bias or social desirability bias. The present study utilized a global coping score derived from the Brief-COPE. The present study also employed a global coping score derived from the Brief-COPE. Because coping encompasses both adaptive and maladaptive strategies, the use of an overall Brief-COPE score may have obscured the distinct relationships that different coping dimensions have with professional commitment. Future research is encouraged to examine Brief-COPE subdimensions separately to clarify their unique mediating roles and enhance the precision of interpretation.

**Location revised:** Limitations section

**Comment 3: Please revise the Data Availability Statement. It refers to the privacy of “patients,” but the** **participants were nurses. The institutional process for requesting data access should also be stated more clearly.**

**Response:** We appreciate the reviewer identifying this inconsistency. The Data Availability Statement has been revised to replace “patients” with “participants” (clinical nurses). We also clarified the institutional procedure for requesting access to the de-identified dataset, including contact information for the Institutional Review Board and the corresponding author.

**Revised content:** The data underlying this study cannot be made publicly available because they contain potentially identifiable information related to the participating nurses, and public sharing could compromise participant confidentiality. Researchers who meet the criteria for access to confidential data may submit a request to the Institutional Review Board of Chung Shan Medical University Hospital for consideration.

**Location revised:** Data Availability Statement

**Comment 4: A final English language revision is recommended to correct minor grammatical and typographical errors.**

**Response:** Thank you for this recommendation. The manuscript has undergone an additional English language review. Minor grammatical errors, typographical mistakes, and wording inconsistencies were corrected throughout the manuscript to improve readability and clarity.

**Response to Reviewer #2**

**Comment: The manuscript addresses a relevant and important topic for the field and has the potential to contribute meaningfully to the existing literature. The authors have adequately addressed the comments raised during the previous round of review and have implemented the requested revisions. However, in this second review, one methodological issue remains that I believe should be clarified prior to publication. There appears to be an inconsistency in the reporting of the sample size calculation. The manuscript states that the G*Power analysis indicated a minimum required sample of 130 participants, which was increased to 156 after accounting for a 20% attrition rate. However, the study ultimately recruited 355 participants, of whom 345 completed the questionnaires. This final sample is more than double the calculated minimum requirement.**

**Therefore, I recommend that the authors clarify the rationale for recruiting 355 participants when the estimated minimum sample size was 156. This discrepancy is not discussed in the manuscript and may raise questions regarding the appropriateness of the reported power analysis. Furthermore, it would be helpful to provide additional details regarding the parameters used in G*Power and their correspondence to the analytical model employed. Given that the study involves a serial mediation model with one independent variable, two mediators, and covariates, the current description may lead readers to infer that the power analysis was based on a standard multiple linear regression model rather than the mediation framework actually used. Clarification of this issue would strengthen the methodological transparency and rigor of the study.**

**Response:** We sincerely thank the reviewer for this thoughtful observation and valuable suggestion. We agree that additional clarification regarding the sample size estimation and the relationship between the G*Power analysis and the serial mediation framework would improve the methodological transparency of the study.

First, the sample size was calculated using software, the G*Power version 3.1 [16], with the following settings: statistical test (linear multiple regression), medium effect size (0.15), significance level (0.05), statistical power (0.95). The results indicated that a minimum of 130 participants were required. Considering a 20% potential data loss rate, the final target sample size was set at 156 participants.

Second, although 156 participants were considered sufficient to satisfy the minimum statistical requirements, the study employed convenience sampling and sought to recruit all eligible nurses who were available during the data collection period. Consequently, 355 nurses agreed to participate, and 345 valid questionnaires were retained for analysis. Recruiting a larger sample than the minimum required is consistent with recommendations to enhance statistical precision, reduce sampling error, and improve the stability of indirect effect estimates in mediation analyses.

Third, we acknowledge that the serial mediation analysis was subsequently conducted using PROCESS Model 6. Because no universally accepted a priori sample size estimation procedure exists for PROCESS-based serial mediation models, the G*Power calculation served as a conservative estimate of the minimum sample size required under a multiple regression framework. The final sample size substantially exceeded this threshold, thereby providing adequate statistical power for the mediation analyses performed.

Accordingly, we have revised the Methods section to clarify the rationale for the final sample size and expanded the description of the G*Power parameters and their correspondence to the analytical approach used in this study.

**Added text:** Nevertheless, all eligible nurses available during the study period were invited to participate using convenience sampling to maximize statistical precision and improve the stability of mediation estimates. A total of 355 nurses agreed to participate, and 345 valid questionnaires were ultimately included in the final analyses, yielding a valid response rate of 97.2%. The final sample size substantially exceeded the minimum requirement and provided adequate statistical power for the serial mediation analyses conducted using PROCESS Model 6.

**Location revised:** Methods: Design and Participants

**Added text**: In addition, although the a priori sample size calculation was based on a multiple regression framework rather than a specific serial mediation model, the final sample substantially exceeded the estimated minimum requirement, thereby enhancing the precision and stability of the mediation estimates.

**Location revised:** Limitations section

**Response to Reviewer #3 and #4**

**Revisions made in the manuscript:** Reviewer #3 and #4 did not request any further revisions in this review round.

**Response:** Therefore, no additional modifications were made to the manuscript. The authors respectfully acknowledge the reviewer's positive evaluation of the revised manuscript and appreciate the constructive feedback provided throughout the peer-review process.

We sincerely appreciate the reviewer’s insightful comments, which have helped improve the clarity and overall quality of the manuscript.

Sincerely,

Chia-En Hsieh, RN, NP, Assistant Professor, PhD.

Liver transplantation center, Chung Shan Medical University Hospital, Taichung, Taiwan

e-Mail : chiaen69918@gmail.com
