# Supplementary material for: The relationship between nurse stress and professional commitment in clinical nurses: A serial multiple mediation model through resilience and coping
Source: PLoS One. 2026 Aug 24;21(8):e0356880. doi: 10.1371/journal.pone.0356880 (PMC13502600; doi:10.1371/journal.pone.0356880)
Supplement: Supplementary file 4 [file pone.0356880.s005.docx]

**Dear Academic Editor and Reviewer's**

**All revisions made in response to the reviewers' and editor's comments are highlighted in yellow in the revised manuscript. The highlighted text indicates the sections that have been modified, added, or clarified.**

**Response to Reviewer #1**

Thank you very much for your positive evaluation of our revised manuscript and for recognizing the improvements in the clarity of the study design, sample description, mediation analysis, and acknowledgment of the cross-sectional nature of the study. We sincerely appreciate your constructive suggestions. We have carefully revised the manuscript accordingly. Detailed responses to each comment are provided below.

**Comment 1: The authors have satisfactorily addressed the main methodological concerns raised during the previous rounds of review, and the manuscript has been substantially improved. However, causal language remains in the abstract, particularly in the statement that nurse stress “negatively affected” professional commitment and that resilience and coping strategies may “mitigate this impact.” Given the cross-sectional design of the study, these expressions should be replaced with wording based on associations between the variables. The authors should also correct the remaining grammatical repetitions.**

**Response:** We sincerely thank the reviewer for the positive evaluation of our revised manuscript and for the helpful comments. In response to the reviewer's suggestion, we have further revised the Abstract to eliminate the remaining causal language and ensure consistency with the cross-sectional design of the study. Specifically, expressions such as "negatively affected" and "mitigate this impact" have been replaced with wording that emphasizes associations rather than causal relationships. In addition, we carefully proofread the entire manuscript and corrected the remaining grammatical repetitions and minor language issues to improve clarity and readability. These revisions further strengthen the methodological consistency and overall quality of the manuscript.

**Revised content:** Higher nurse stress was significantly associated with lower professional commitment. Resilience and coping were significantly associated with this relationship as serial mediating factors. Given the cross-sectional design, these findings should be interpreted as associations rather than causal effects. Strengthening resilience and adaptive coping may help support nurses' professional commitment.

**Location revised:** Abstract section, conclusion.

**Revised content:** To test the study hypotheses (H1, H2, and H3), serial mediation analysis was performed using PROCESS macro Model 6 (version 4.2) developed by Hayes.

**Location revised:** Methods section, statistical analysis.

**Revised content:** The present study employed a global coping score derived from the Brief-COPE. Because the Brief-COPE includes both adaptive and maladaptive coping strategies, the overall score may have obscured the distinct relationships between specific coping dimensions and professional commitment.

**Location revised:** Limitations

**Response to Reviewer #2**

We sincerely thank the reviewer for the positive evaluation of our manuscript and for recognizing the methodological improvements made during the revision process. We appreciate the reviewer’s careful reading of the manuscript and have addressed all remaining editorial comments as follows.

**Comment 1: Verify that the Abstract consistently uses language reflecting association rather than causation, in line with the revisions already implemented in the main text. At present, expressions such as "had a significant negative total effect" and "negatively affected" may still be interpreted as implying causality.**

**Response:** Thank you for this important suggestion. We agree that the Abstract should fully reflect the limitations of the cross-sectional design. Accordingly, we revised the Abstract to remove the remaining causal expressions.

Specifically,

- "had a significant negative total effect" has been revised to "was significantly negatively associated with professional commitment."
- "Nurse stress negatively affected professional commitment" has been revised to "Higher nurse stress was significantly associated with lower professional commitment."
- We also added wording emphasizing that the findings should be interpreted as associations rather than causal relationships.

These revisions ensure consistency between the Abstract and the main text.

**Revised content:** Nurse stress was significantly negatively associated with professional commitment (total association: c = −0.0808, p < 0.001).

**Location revised:** Abstract section, results.

**Revised content:** Higher nurse stress was significantly associated with lower professional commitment. Resilience and coping were significantly associated with this relationship as serial mediating factors. Given the cross-sectional design, these findings should be interpreted as associations rather than causal effects. Strengthening resilience and adaptive coping may help support nurses' professional commitment.

**Location revised:** Abstract section, conclusion.

**Comment 2 : Correct the editorial issues identified, including the duplicated sentence, and the residual text fragment language throughout the manuscript.**

**Response:** Thank you for identifying these editorial issues. We have carefully proofread the entire manuscript and made the following revisions:

- Removed duplicated sentences and residual text fragments.
- Corrected minor grammatical and typographical errors throughout the manuscript.

**Revised content:** The Nurses’ Professional Commitment Scale (NPCS) was developed by Lin et al. (2007).

**Location revised:** Methods section, measures.

**Revised content:** To test the study hypotheses (H1, H2, and H3), serial mediation analysis was performed using PROCESS macro Model 6 (version 4.2) developed by Hayes.

**Location revised:** Methods section, statistical analysis.

**Revised content:** The present study employed a global coping score derived from the Brief-COPE. Because the Brief-COPE includes both adaptive and maladaptive coping strategies, the overall score may have obscured the distinct relationships between specific coping dimensions and professional commitment.

**Location revised:** Limitations

**Comment 3 : Verify references 35 and 36, as there appears to be an inconsistency between the publication year listed (2026) and the year embedded in the DOI (2025).**

**Response :** Thank you for carefully checking the references.

**References :** 35. Bartoszuk J, Sierakowska M. Selected aspects of the psychosocial functioning of medical personnel of emergency medical teams-a cross-sectional study. Front Public Health. 2026;13:1754424. https://doi.org/10.3389/fpubh.2025.1754424

**References :** 36. Song L, Du L, Xu L, Wu M, Li X. Navigating Challenges and Building Resilience: Qualitative Insights into Disaster Nursing Preparedness in Chinese Non-First-Tier Cities. Disaster Med Public Health Prep. 2025;19:e250. https://doi.org/10.1017/dmp.2025.10177

We sincerely appreciate the reviewer’s insightful comments, which have helped improve the clarity and overall quality of the manuscript.

Sincerely,

Chia-En Hsieh, RN, NP, Assistant Professor, PhD.

Liver transplantation center, Chung Shan Medical University Hospital, Taichung, Taiwan

e-Mail : chiaen69918@gmail.com
