# Supplementary material for: The relationship between nurse stress and professional commitment in clinical nurses: A serial multiple mediation model through resilience and coping
Source: PLoS One. 2026 Aug 24;21(8):e0356880. doi: 10.1371/journal.pone.0356880 (PMC13502600; doi:10.1371/journal.pone.0356880)
Supplement: Peer review history

# Peer review: Decision Letter 0

**DOI:** 10.1371/journal.pone.0356880.r001

**Authors:** Ahmed El-Sayed

29 Apr 2026

Dear Author

Thank you for submitting your manuscript to PLOS ONE. After careful consideration, we feel that it has merit but does not fully meet PLOS ONE’s publication criteria as it currently stands. Therefore, we invite you to submit a revised version of the manuscript that addresses the points raised during the review process.

Please submit your revised manuscript by Jun 13 2026 11:59PM. If you will need more time than this to complete your revisions, please reply to this message or contact the journal office at plosone@plos.org. When you're ready to submit your revision, log on to https://www.editorialmanager.com/pone/ and select the 'Submissions Needing Revision' folder to locate your manuscript file.

- A letter that responds to each point raised by the academic editor and reviewer(s). You should upload this letter as a separate file labeled 'Response to Reviewers'.
- A marked-up copy of your manuscript that highlights changes made to the original version. You should upload this as a separate file labeled 'Revised Manuscript with Track Changes'.
- An unmarked version of your revised paper without tracked changes. You should upload this as a separate file labeled 'Manuscript'.

If you would like to make changes to your financial disclosure, please include your updated statement in your cover letter. Guidelines for resubmitting your figure files are available below the reviewer comments at the end of this letter.

If applicable, we recommend that you deposit your laboratory protocols in protocols.io to enhance the reproducibility of your results. Protocols.io assigns your protocol its own identifier (DOI) so that it can be cited independently in the future. For instructions see: https://journals.plos.org/plosone/s/submission-guidelines#loc-laboratory-protocols. Additionally, PLOS ONE offers an option for publishing peer-reviewed Lab Protocol articles, which describe protocols hosted on protocols.io. Read more information on sharing protocols at https://plos.org/protocols?utm_medium=editorial-email&utm_source=authorletters&utm_campaign=protocols.

As the corresponding author, your ORCID iD is verified in the submission system and will appear in the published article. PLOS supports the use of ORCID, and we encourage all coauthors to register for an ORCID iD and use it as well. Please encourage your coauthors to verify their ORCID iD within the submission system before final acceptance, as unverified ORCID iDs will not appear in the published article. *Only*  the individual author can complete the verification step; PLOS staff *cannot*  verify ORCID iDs on behalf of authors.

We look forward to receiving your revised manuscript.

Kind regards,

Ahmed Abdelwahab Ibrahim El-Sayed

Academic Editor

PLOS One

Journal Requirements:

When submitting your revision, we need you to address these additional requirements.

1. Please ensure that your manuscript meets PLOS ONE's style requirements, including those for file naming. The PLOS ONE style templates can be found at

https://journals.plos.org/plosone/s/file?id=wjVg/PLOSOne_formatting_sample_main_body.pdf and

https://journals.plos.org/plosone/s/file?id=ba62/PLOSOne_formatting_sample_title_authors_affiliations.pdf.

2. Please include your tables as part of your main manuscript and remove the individual files. Please note that supplementary tables (should remain/ be uplo`aded) as separate "supporting information" files.

3. We note that you have indicated that there are restrictions to data sharing for this study. For studies involving human research participant data or other sensitive data, we encourage authors to share de-identified or anonymized data. However, when data cannot be publicly shared for ethical reasons, we allow authors to make their data sets available upon request. For information on unacceptable data access restrictions, please see http://journals.plos.org/plosone/s/data-availability#loc-unacceptable-data-access-restrictions.

Before we proceed with your manuscript, please address the following prompts:

a) If there are ethical or legal restrictions on sharing a de-identified data set, please explain them in detail (e.g., data contain potentially identifying or sensitive patient information, data are owned by a third-party organization, etc.) and who has imposed them (e.g., a Research Ethics Committee or Institutional Review Board, etc.). Please also provide contact information for a data access committee, ethics committee, or other institutional body to which data requests may be sent.

b) If there are no restrictions, please upload the minimal anonymized data set necessary to replicate your study findings to a stable, public repository and provide us with the relevant URLs, DOIs, or accession numbers. Please see http://www.bmj.com/content/340/bmj.c181.long for guidelines on how to de-identify and prepare clinical data for publication. For a list of recommended repositories, please see https://journals.plos.org/plosone/s/recommended-repositories. You also have the option of uploading the data as Supporting Information files, but we would recommend depositing data directly to a data repository if possible.

Please update your Data Availability statement in the submission form accordingly.

4. In this instance it seems there may be acceptable restrictions in place that prevent the public sharing of your minimal data. However, in line with our goal of ensuring long-term data availability to all interested researchers, PLOS’ Data Policy states that authors cannot be the sole named individuals responsible for ensuring data access (http://journals.plos.org/plosone/s/data-availability#loc-acceptable-data-sharing-methods).

Data requests to a non-author institutional point of contact, such as a data access or ethics committee, helps guarantee long term stability and availability of data. Providing interested researchers with a durable point of contact ensures data will be accessible even if an author changes email addresses, institutions, or becomes unavailable to answer requests.

Before we proceed with your manuscript, please also provide non-author contact information (phone/email/hyperlink) for a data access committee, ethics committee, or other institutional body to which data requests may be sent. If no institutional body is available to respond to requests for your minimal data, please consider if there any institutional representatives who did not collaborate in the study, and are not listed as authors on the manuscript, who would be able to hold the data and respond to external requests for data access? If so, please provide their contact information (i.e., email address). Please also provide details on how you will ensure persistent or long-term data storage and availability.

5. Your ethics statement should only appear in the Methods section of your manuscript. If your ethics statement is written in any section besides the Methods, please move it to the Methods section and delete it from any other section. Please ensure that your ethics statement is included in your manuscript, as the ethics statement entered into the online submission form will not be published alongside your manuscript.

6. If the reviewer comments include a recommendation to cite specific previously published works, please review and evaluate these publications to determine whether they are relevant and should be cited. There is no requirement to cite these works unless the editor has indicated otherwise.

Additional Editor Comments

Dear Authors,

Thank you for your submission to PLOS ONE. The review of your submission has now been completed. The reviewer has raised several issues that need to be carefully considered and addressed before we can consider the manuscript further.

[Note: HTML markup is below. Please do not edit.]

Reviewers' comments:

Reviewer's Responses to Questions

**Comments to the Author**

1. Is the manuscript technically sound, and do the data support the conclusions?

Reviewer #1: Partly

Reviewer #2: Partly

Reviewer #3: Partly

Reviewer #4: Yes

**********

2. Has the statistical analysis been performed appropriately and rigorously?

Reviewer #1: Yes

Reviewer #2: No

Reviewer #3: No

Reviewer #4: Yes

**********

3. Have the authors made all data underlying the findings in their manuscript fully available?

The PLOS Data policy

Reviewer #1: No

Reviewer #2: Yes

Reviewer #3: No

Reviewer #4: Yes

**********

4. Is the manuscript presented in an intelligible fashion and written in standard English?

Reviewer #1: Yes

Reviewer #2: Yes

Reviewer #3: Yes

Reviewer #4: Yes

**********

Reviewer #1: The topic is relevant, and the study addresses an important issue in the field of clinical nursing. However, in its current form, the manuscript requires substantial revision before it can be considered for publication.

First, the overall presentation of the study would benefit from greater clarity and consistency across sections. In particular, some aspects related to the sample and setting are not described in a fully consistent manner throughout the manuscript, and this point should be carefully revised to make the study design and recruitment context entirely clear to readers.

Second, although the general analytical approach is understandable, there are methodological and data analysis issues that require further revision and clarification. I would encourage the authors to reconsider this section carefully and ensure that the reporting of the analytical strategy is sufficiently transparent, coherent, and aligned with the study objectives.

Third, the interpretation of the findings should be more cautious. Because this is a cross-sectional study conducted in a single center, some parts of the manuscript use language that appears stronger than warranted by the study design. The wording in the Discussion, and especially in the Conclusion, should be revised to avoid causal implications and to better reflect an associative interpretation of the findings.

In addition, the conclusion is currently too brief and somewhat generic. It should summarize the main findings of the study more clearly, while remaining proportionate to the evidence presented.

Overall, the manuscript addresses a meaningful topic and has potential, but substantial revision is needed to improve clarity, methodological transparency, internal consistency, and balance in the conclusions.

Reviewer #2: Dear Authors,

The manuscript addresses a relevant research question and employs validated instruments appropriate for the study context. However, it presents several methodological issues that should be addressed before the manuscript can be considered for publication.

Regarding the keywords, it would be important to broaden them to better reflect the range of professions included in the study, as currently only two are explicitly mentioned. Including a more general term or all relevant professional groups would improve indexing and visibility.

In the abstract, it is recommended to structure the content clearly by sections and to include key methodological details such as sample sizes and response rates. Additionally, the main statistical findings should be reported (e.g., effect sizes or key coefficients). It is also essential to clarify that this is not a longitudinal follow-up of the same individuals, but rather repeated cross-sectional samples.

Concerning the hypothesis, its current formulation does not allow for a clear evaluation or operationalization. Furthermore, it is not evident how the results support or refute it. I suggest reformulating the hypothesis more explicitly, ensuring alignment with the theoretical framework and specifying the expected direction of associations.

Although the manuscript presents a solid and up-to-date theoretical background, it lacks a clearly defined research gap. It would be important to explicitly state why this study is necessary and what specific contribution it makes to existing literature. In addition, several associations are described (e.g., marital status and occupational balance), but their relevance is not sufficiently justified. The authors should clarify how this study contributes to confirming, challenging, or expanding current evidence.

The manuscript would also benefit from a clearer and more precise conceptual definition of occupational balance, as this is a central variable in the study. A stronger conceptual grounding would enhance the reader’s understanding and interpretation of the findings.

Regarding the methods, it is crucial to explicitly state that the study does not follow the same individuals over time but instead consists of independent cross-sectional samples. The sampling process is not described with sufficient clarity or detail, which may compromise the validity of the findings. The authors should specify the sampling strategy (convenience or probabilistic sampling), the size of the target population, the number of individuals invited, and the number who declined participation. Without this information, it is not possible to assess the risk of non-response bias.

Additionally, the use of univariate and multivariate linear regression analyses with OBQ as a continuous outcome is appropriate in principle; however, there is no report of verification of statistical assumptions (normality, multicollinearity). It is also unclear whether a statistical power calculation was performed, or whether the sample size is sufficient, particularly for subgroup analyses.

In the results section, the baseline characteristics table (Table 1) is presented only in the Supporting Information rather than in the main text. This is essential information that should be included in the main manuscript to allow readers to assess the representativeness of the sample.

Furthermore, the finding that living alone is associated with higher OBQ scores at year three (Y3) is not adequately discussed or interpreted. It would be important to provide a clearer explanation of the direction and potential meaning of this association.

In the discussion, the section addressing age differences appears somewhat speculative and is not sufficiently grounded in the specific statistical results from the subgroup analyses. Strengthening the link between the findings and their interpretation would improve the scientific rigor of this section.

Finally, in the conclusion, the statement that “occupational balance appears to function as a protective factor” is not supported by the study design. Given the cross-sectional nature of the data, causal inferences or directional claims cannot be made. This statement should be revised to reflect association rather than causal relationships.

I hope that these comments are helpful and constructive in strengthening the manuscript. The topic addressed is both relevant and timely, and with further clarification and methodological refinement, the study has the potential to make a meaningful contribution to the field. I encourage the authors to consider these suggestions carefully to enhance the clarity, rigor, and overall impact of their work.

Reviewer #3: Dear Authors:

The manuscript addresses a highly relevant and timely topic in nursing research. The use of a serial multiple mediation model to analyze the psychological mechanisms linking stress to professional commitment is analytically sophisticated and provides valuable insights for workforce retention strategies. However, there are significant methodological and conceptual limitations that require clarification to ensure the rigor of the findings:

•Brief-COPE Aggregation is a critical point because the authors utilize the Brief-COPE to create a single global "coping" score. This approach is methodologically debatable. The Brief-COPE assesses 14 distinct strategies that are theoretically and clinically divergent. By aggregating adaptive strategies (e.g., planning, active coping) with maladaptive ones (e.g., substance use, denial, behavioral disengagement), the model obscures the unique impact of each strategy. I suggest that the authors perform a complementary analysis grouping items into Adaptive and Maladaptive subscales. This would allow the model to demonstrate whether the impact of stress on professional commitment is mediated differently by each type of strategy, offering more precise intervention recommendations for nursing management.

•While the use of a convenience sample is common in clinical research, the authors must expand the discussion on the limitations of this approach. Specifically, how do the characteristics of a single large medical center influence the results compared to smaller community hospitals? This is vital for understanding the generalizability of the serial mediation model.

•The authors must include a formal "Data Availability Statement," indicating exactly where the raw data will be accessible to the scientific community.

•The use of PROCESS Model 6 is appropriate. However, the authors should explicitly state why this specific model was chosen over alternative structural equation modeling (SEM) approaches, particularly given the serial nature of the mediators.

•The results confirm a partial mediation model, as the direct effect remains significant. The authors should further explore the "unexplained" portion of the direct effect. What other factors—perhaps organizational-level variables beyond individual resilience—might be driving the decrease in commitment among nurses?

•While the suggestions regarding mindfulness and emotional regulation are sound, they must be balanced with a discussion on system or organizational changes. Nursing commitment is not solely an individual psychological trait but is deeply influenced by workload, safe staffing, and institutional support.

Finally, I have some suggestions:

•Ensure that all references are updated to reflect the most current literature (2024–2026) regarding nurse retention and mental health post-pandemic.

•And please verify that the mediation model diagram (Figure 1) explicitly labels the coefficients with their respective significance levels and that the text consistently references these values.

Reviewer #4: Minor grammatical errors need correcting. Regarding the Limitations section - please provide greater clarity and examples of limitations and study results do not imply causal relationships. Consider restructuring sentences regarding confirmation of hypotheses 1, 2, and 3 to introduce hypotheses again prior to confirmation from the data.

**********

what does this mean?). If published, this will include your full peer review and any attached files.

If you choose “no”, your identity will remain anonymous but your review may still be made public.

**Do you want your identity to be public for this peer review?**  For information about this choice, including consent withdrawal, please see our Privacy Policy

Reviewer #1: **Yes:** Federico Cucci

Reviewer #2: No

Reviewer #3: **Yes:** Katya Cuadros-Carlesi

Reviewer #4: No

**********

[NOTE: If reviewer comments were submitted as an attachment file, they will be attached to this email and accessible via the submission site. Please log into your account, locate the manuscript record, and check for the action link "View Attachments". If this link does not appear, there are no attachment files.]

To ensure your figures meet our technical requirements, please review our figure guidelines: https://journals.plos.org/plosone/s/figures

You may also use PLOS’s free figure tool, NAAS, to help you prepare publication quality figures: https://journals.plos.org/plosone/s/figures#loc-tools-for-figure-preparation.

NAAS will assess whether your figures meet our technical requirements by comparing each figure against our figure specifications.

Attachment Submitted filename: PONE-D-26-11739_Patricia Brothers27APR2026.pdf

# Author comment: Author response to Decision Letter 1

**DOI:** 10.1371/journal.pone.0356880.r002

2 Jun 2026

Dear Academic Editor and Reviewer's

We would like to submit the enclosed manuscript for possible publication in PLoS One. The manuscript is entitled "The Relationship Between Nurse Stress and Professional Commitment in Clinical Nurses: A Serial Multiple Mediation Model Through Resilience and Coping."

All revisions made in response to the reviewers' and editor's comments are highlighted in yellow in the revised manuscript. The highlighted text indicates the sections that have been modified, added, or clarified. We sincerely appreciate the reviewer’s insightful comments, which have helped improve the clarity and overall quality of the manuscript.

Sincerely,

Chia-En Hsieh, RN, NP, Assistant Professor, PhD.

Liver transplantation center, Chung Shan Medical University Hospital, Taichung, Taiwan

e-Mail : chiaen69918@gmail.com

Attachment Submitted filename: Response to Reviewer 20260601.docx

# Peer review: Decision Letter 1

**DOI:** 10.1371/journal.pone.0356880.r003

**Authors:** Ahmed El-Sayed

19 Jun 2026

Dear Dr. Hsieh,

Thank you for submitting your manuscript to PLOS ONE. After careful consideration, we feel that it has merit but does not fully meet PLOS ONE’s publication criteria as it currently stands. Therefore, we invite you to submit a revised version of the manuscript that addresses the points raised during the review process.

Please submit your revised manuscript by Aug 03 2026 11:59PM. If you will need more time than this to complete your revisions, please reply to this message or contact the journal office at plosone@plos.org. When you're ready to submit your revision, log on to https://www.editorialmanager.com/pone/ and select the 'Submissions Needing Revision' folder to locate your manuscript file.

- A letter that responds to each point raised by the academic editor and reviewer(s). You should upload this letter as a separate file labeled 'Response to Reviewers'.
- A marked-up copy of your manuscript that highlights changes made to the original version. You should upload this as a separate file labeled 'Revised Manuscript with Track Changes'.
- An unmarked version of your revised paper without tracked changes. You should upload this as a separate file labeled 'Manuscript'.

If you would like to make changes to your financial disclosure, please include your updated statement in your cover letter. Guidelines for resubmitting your figure files are available below the reviewer comments at the end of this letter.

If applicable, we recommend that you deposit your laboratory protocols in protocols.io to enhance the reproducibility of your results. Protocols.io assigns your protocol its own identifier (DOI) so that it can be cited independently in the future. For instructions see: https://journals.plos.org/plosone/s/submission-guidelines#loc-laboratory-protocols. Additionally, PLOS ONE offers an option for publishing peer-reviewed Lab Protocol articles, which describe protocols hosted on protocols.io. Read more information on sharing protocols at https://plos.org/protocols?utm_medium=editorial-email&utm_source=authorletters&utm_campaign=protocols.

As the corresponding author, your ORCID iD is verified in the submission system and will appear in the published article. PLOS supports the use of ORCID, and we encourage all coauthors to register for an ORCID iD and use it as well. Please encourage your coauthors to verify their ORCID iD within the submission system before final acceptance, as unverified ORCID iDs will not appear in the published article. *Only*  the individual author can complete the verification step; PLOS staff *cannot*  verify ORCID iDs on behalf of authors.

We look forward to receiving your revised manuscript.

Kind regards,

Ahmed Abdelwahab Ibrahim El-Sayed

Academic Editor

PLOS One

Journal Requirements:

If the reviewer comments include a recommendation to cite specific previously published works, please review and evaluate these publications to determine whether they are relevant and should be cited. There is no requirement to cite these works unless the editor has indicated otherwise.

Please review your reference list to ensure that it is complete and correct. If you have cited papers that have been retracted, please include the rationale for doing so in the manuscript text, or remove these references and replace them with relevant current references. Any changes to the reference list should be mentioned in the rebuttal letter that accompanies your revised manuscript. If you need to cite a retracted article, indicate the article’s retracted status in the References list and also include a citation and full reference for the retraction notice.

**Additional Editor Comments**

Dear Authors,

We would like to thank you for their efforts in revising the manuscript. I have completed my review of the revised version, and the authors have addressed most of the previous comments satisfactorily. However, some minor issues remain that should be considered and addressed before a final editorial decision regarding this study is made.

Overall, the manuscript has improved considerably, and I believe that addressing these minor points will further strengthen the work.

[Note: HTML markup is below. Please do not edit.]

Reviewers' comments:

Reviewer's Responses to Questions

**Comments to the Author**

Reviewer #1: All comments have been addressed

Reviewer #2: All comments have been addressed

Reviewer #3: All comments have been addressed

Reviewer #4: All comments have been addressed

**********

2. Is the manuscript technically sound, and do the data support the conclusions?

Reviewer #1: Partly

Reviewer #2: Yes

Reviewer #3: Yes

Reviewer #4: Yes

**********

3. Has the statistical analysis been performed appropriately and rigorously?

Reviewer #1: N/A

Reviewer #2: Yes

Reviewer #3: Yes

Reviewer #4: Yes

**********

4. Have the authors made all data underlying the findings in their manuscript fully available?

The PLOS Data policy

Reviewer #1: No

Reviewer #2: No

Reviewer #3: Yes

Reviewer #4: Yes

**********

5. Is the manuscript presented in an intelligible fashion and written in standard English?

Reviewer #1: No

Reviewer #2: Yes

Reviewer #3: Yes

Reviewer #4: Yes

**********

Reviewer #1: The revised manuscript addresses a relevant topic and has improved in clarity. The study design, sample, and mediation analysis are now more clearly described, and the authors appropriately acknowledge the cross-sectional nature of the study. The manuscript is generally suitable for publication after minor revisions.

Before acceptance, I suggest the following minor revisions:

- Please further reduce causal language throughout the manuscript, especially in the Discussion and Conclusion. Terms such as “impact,” “influences,” and “effects” should be replaced, where appropriate, with more cautious wording such as “association” or “is associated with.”

- Please clarify the rationale for using a global Brief-COPE score, since this instrument includes both adaptive and maladaptive coping strategies. If no further analyses are performed, this limitation should be emphasized more clearly.

- Please revise the Data Availability Statement. It refers to the privacy of “patients,” but the participants were nurses. The institutional process for requesting data access should also be stated more clearly.

- A final English language revision is recommended to correct minor grammatical and typographical errors.

Overall, the remaining issues concern clarity, reporting, and interpretation rather than the overall validity of the study. I therefore recommend minor revision

Reviewer #2: The manuscript addresses a relevant and important topic for the field and has the potential to contribute meaningfully to the existing literature. The authors have adequately addressed the comments raised during the previous round of review and have implemented the requested revisions.

However, in this second review, one methodological issue remains that I believe should be clarified prior to publication.

There appears to be an inconsistency in the reporting of the sample size calculation. The manuscript states that the G*Power analysis indicated a minimum required sample of 130 participants, which was increased to 156 after accounting for a 20% attrition rate. However, the study ultimately recruited 355 participants, of whom 345 completed the questionnaires. This final sample is more than double the calculated minimum requirement.

Therefore, I recommend that the authors clarify the rationale for recruiting 355 participants when the estimated minimum sample size was 156. This discrepancy is not discussed in the manuscript and may raise questions regarding the appropriateness of the reported power analysis. Furthermore, it would be helpful to provide additional details regarding the parameters used in G*Power and their correspondence to the analytical model employed. Given that the study involves a serial mediation model with one independent variable, two mediators, and covariates, the current description may lead readers to infer that the power analysis was based on a standard multiple linear regression model rather than the mediation framework actually used. Clarification of this issue would strengthen the methodological transparency and rigor of the study.

Reviewer #3: Dear Authors,

I have thoroughly reviewed the new version of the article: "The relationship between nurse stress and professional commitment in clinical nurses: A serial multiple mediation model through resilience and coping” and my observations are as follows:

• Methodological Transparency & Statistical Robustness: The statistical strategy section has been substantially elevated. The rationale behind choosing Hayes' PROCESS Macro Model 6 over alternative latent variable modeling is clearly justified, and the data distribution tracking has been adequately declared. Furthermore, path coefficients, bootstrapping intervals, and significance thresholds are now impeccably standardized and cross-referenced with the legend in Figure 1.

• Linguistic Precision & Mitigation of Overinterpretation: In alignment with the constraints of observational research designs, all overstatements and causal terminology have been systematically replaced with precise associative and correlational language.

• Analytical Depth and Balancing of Interventions: The discussion section has been balanced to look beyond individual-level traits. By framing the unexplained direct path through systemic and organizational variables—such as staffing adequacy, workload intensity, and workplace culture—the paper provides a much more holistic and applicable explanatory model for global nursing workforce retention policies.

• Data Transparency & Bibliographic Rigor: The reference list has been thoroughly updated to encompass recent advancements in the field (2024–2026), and a clear, journal-compliant Data Availability Statement under specific IRB confidentiality constraints has been established.

I congratulate the authors for considering the observations of each of the reviewers, because the manuscript now stands as a technically sound, transparently reported, and meaningful contribution to healthcare management literature.

Reviewer #4: The authors addressed all of the comments for a structured, presentation free of grammatical errors for publication.

**********

what does this mean?). If published, this will include your full peer review and any attached files.

If you choose “no”, your identity will remain anonymous but your review may still be made public.

**Do you want your identity to be public for this peer review?**  For information about this choice, including consent withdrawal, please see our Privacy Policy

Reviewer #1: **Yes:** Federico Cucci

Reviewer #2: No

Reviewer #3: **Yes:** Katya Cuadros-Carlesi

Reviewer #4: No

**********

[NOTE: If reviewer comments were submitted as an attachment file, they will be attached to this email and accessible via the submission site. Please log into your account, locate the manuscript record, and check for the action link "View Attachments". If this link does not appear, there are no attachment files.]

To ensure your figures meet our technical requirements, please review our figure guidelines: https://journals.plos.org/plosone/s/figures

You may also use PLOS’s free figure tool, NAAS, to help you prepare publication quality figures: https://journals.plos.org/plosone/s/figures#loc-tools-for-figure-preparation.

NAAS will assess whether your figures meet our technical requirements by comparing each figure against our figure specifications.

# Author comment: Author response to Decision Letter 2

**DOI:** 10.1371/journal.pone.0356880.r004

23 Jun 2026

Dear Academic Editor and Reviewer's

All revisions made in response to the reviewers' and editor's comments are highlighted in yellow in the revised manuscript. The highlighted text indicates the sections that have been modified, added, or clarified.

We sincerely appreciate the reviewer’s insightful comments, which have helped improve the clarity and overall quality of the manuscript.

Sincerely,

Chia-En Hsieh, RN, NP, Assistant Professor, PhD.

Liver transplantation center, Chung Shan Medical University Hospital, Taichung, Taiwan

e-Mail : chiaen69918@gmail.com

Attachment Submitted filename: Response to Reviewer 20260623.docx

# Peer review: Decision Letter 2

**DOI:** 10.1371/journal.pone.0356880.r005

**Authors:** Ahmed El-Sayed

15 Jul 2026

Dear Dr. Hsieh,

Thank you for submitting your manuscript to PLOS ONE. After careful consideration, we feel that it has merit but does not fully meet PLOS ONE’s publication criteria as it currently stands. Therefore, we invite you to submit a revised version of the manuscript that addresses the points raised during the review process.

Please submit your revised manuscript by Aug 29 2026 11:59PM. If you will need more time than this to complete your revisions, please reply to this message or contact the journal office at plosone@plos.org. When you're ready to submit your revision, log on to https://www.editorialmanager.com/pone/ and select the 'Submissions Needing Revision' folder to locate your manuscript file.

- A letter that responds to each point raised by the academic editor and reviewer(s). You should upload this letter as a separate file labeled 'Response to Reviewers'.
- A marked-up copy of your manuscript that highlights changes made to the original version. You should upload this as a separate file labeled 'Revised Manuscript with Track Changes'.
- An unmarked version of your revised paper without tracked changes. You should upload this as a separate file labeled 'Manuscript'.

If you would like to make changes to your financial disclosure, please include your updated statement in your cover letter. Guidelines for resubmitting your figure files are available below the reviewer comments at the end of this letter.

If applicable, we recommend that you deposit your laboratory protocols in protocols.io to enhance the reproducibility of your results. Protocols.io assigns your protocol its own identifier (DOI) so that it can be cited independently in the future. For instructions see: https://journals.plos.org/plosone/s/submission-guidelines#loc-laboratory-protocols. Additionally, PLOS ONE offers an option for publishing peer-reviewed Lab Protocol articles, which describe protocols hosted on protocols.io. Read more information on sharing protocols at https://plos.org/protocols?utm_medium=editorial-email&utm_source=authorletters&utm_campaign=protocols.

As the corresponding author, your ORCID iD is verified in the submission system and will appear in the published article. PLOS supports the use of ORCID, and we encourage all coauthors to register for an ORCID iD and use it as well. Please encourage your coauthors to verify their ORCID iD within the submission system before final acceptance, as unverified ORCID iDs will not appear in the published article. *Only*  the individual author can complete the verification step; PLOS staff *cannot*  verify ORCID iDs on behalf of authors.

We look forward to receiving your revised manuscript.

Kind regards,

Ahmed Abdelwahab Ibrahim El-Sayed

Academic Editor

PLOS One

Journal Requirements:

If the reviewer comments include a recommendation to cite specific previously published works, please review and evaluate these publications to determine whether they are relevant and should be cited. There is no requirement to cite these works unless the editor has indicated otherwise.

Please review your reference list to ensure that it is complete and correct. If you have cited papers that have been retracted, please include the rationale for doing so in the manuscript text, or remove these references and replace them with relevant current references. Any changes to the reference list should be mentioned in the rebuttal letter that accompanies your revised manuscript. If you need to cite a retracted article, indicate the article’s retracted status in the References list and also include a citation and full reference for the retraction notice.

Additional Editor Comments :

Dear Authors,

Thank you for submitting your revised manuscript.

I have completed my review of the revised version. Overall, the manuscript has improved substantially. However, there are still a few minor grammatical and language issues that should be addressed before we can proceed with the final decision on your manuscript.

I look forward to receiving the revised version.

[Note: HTML markup is below. Please do not edit.]

Reviewers' comments:

Reviewer's Responses to Questions

**Comments to the Author**

Reviewer #1: (No Response)

Reviewer #2: All comments have been addressed

**********

2. Is the manuscript technically sound, and do the data support the conclusions?

Reviewer #1: (No Response)

Reviewer #2: Yes

**********

3. Has the statistical analysis been performed appropriately and rigorously?

Reviewer #1: (No Response)

Reviewer #2: Yes

**********

4. Have the authors made all data underlying the findings in their manuscript fully available?

The PLOS Data policy

Reviewer #1: (No Response)

Reviewer #2: Yes

**********

5. Is the manuscript presented in an intelligible fashion and written in standard English?

Reviewer #1: (No Response)

Reviewer #2: Yes

**********

Reviewer #1: The authors have satisfactorily addressed the main methodological concerns raised during the previous rounds of review, and the manuscript has been substantially improved. However, causal language remains in the abstract, particularly in the statement that nurse stress “negatively affected” professional commitment and that resilience and coping strategies may “mitigate this impact.” Given the cross-sectional design of the study, these expressions should be replaced with wording based on associations between the variables. The authors should also correct the remaining grammatical repetitions.

Reviewer #2: Overall, the manuscript presents a methodologically sound design, an appropriate theoretical framework, and has addressed the comments raised during the previous rounds of peer review in a satisfactory manner. I recommend acceptance after minor revision.

The remaining issues are primarily editorial and do not compromise the scientific quality of the manuscript. The authors are encouraged to:

Correct the editorial issues identified, including the duplicated sentence, the residual text fragment, the inconsistency in reference dates, and the standardization of causal language throughout the manuscript.

Verify that the Abstract consistently uses language reflecting association rather than causation, in line with the revisions already implemented in the main text. At present, expressions such as "had a significant negative total effect" and "negatively affected" may still be interpreted as implying causality.

Verify references 35 and 36, as there appears to be an inconsistency between the publication year listed (2026) and the year embedded in the DOI (2025; e.g., 10.3389/fpubh.2025... and 10.1017/dmp.2025...). If these are ahead-of-print publications, this should be indicated appropriately (e.g., ahead of print, in press, or according to the journal's reference style).

**********

what does this mean?). If published, this will include your full peer review and any attached files.

If you choose “no”, your identity will remain anonymous but your review may still be made public.

**Do you want your identity to be public for this peer review?**  For information about this choice, including consent withdrawal, please see our Privacy Policy

Reviewer #1: **Yes:** Federico Cucci

Reviewer #2: No

**********

[NOTE: If reviewer comments were submitted as an attachment file, they will be attached to this email and accessible via the submission site. Please log into your account, locate the manuscript record, and check for the action link "View Attachments". If this link does not appear, there are no attachment files.]

To ensure your figures meet our technical requirements, please review our figure guidelines: https://journals.plos.org/plosone/s/figures

You may also use PLOS’s free figure tool, NAAS, to help you prepare publication quality figures: https://journals.plos.org/plosone/s/figures#loc-tools-for-figure-preparation.

NAAS will assess whether your figures meet our technical requirements by comparing each figure against our figure specifications.

# Author comment: Author response to Decision Letter 3

**DOI:** 10.1371/journal.pone.0356880.r006

20 Jul 2026

Dear Academic Editor and Reviewer's

All revisions made in response to the reviewers' and editor's comments are highlighted in yellow in the revised manuscript. The highlighted text indicates the sections that have been modified, added, or clarified.

We sincerely appreciate the reviewer’s insightful comments, which have helped improve the clarity and overall quality of the manuscript.

Sincerely,

Chia-En Hsieh, RN, NP, Assistant Professor, PhD.

Liver transplantation center, Chung Shan Medical University Hospital, Taichung, Taiwan

e-Mail : chiaen69918@gmail.com

Attachment Submitted filename: Response to Reviewer 20260720.docx

# Editor report: Decision Letter 3

**DOI:** 10.1371/journal.pone.0356880.r007

**Authors:** Ahmed El-Sayed

10 Aug 2026

The relationship between nurse stress and professional commitment in clinical nurses: A serial multiple mediation model through resilience and coping

PONE-D-26-11739R3

Dear Author,

We’re pleased to inform you that your manuscript has been judged scientifically suitable for publication and will be formally accepted for publication once it meets all outstanding technical requirements.

Within one week, you’ll receive an e-mail detailing the required amendments. When these have been addressed, you’ll receive a formal acceptance letter and your manuscript will be scheduled for publication.

An invoice will be generated when your article is formally accepted. Please note, if your institution has a publishing partnership with PLOS and your article meets the relevant criteria, all or part of your publication costs will be covered. Please make sure your user information is up-to-date by logging into Editorial Manager at Editorial Manager® and clicking the ‘Update My Information' link at the top of the page. For questions related to billing, please contact billing support.

If your institution or institutions have a press office, please notify them about your upcoming paper to help maximize its impact. If they’ll be preparing press materials, please inform our press team as soon as possible -- no later than 48 hours after receiving the formal acceptance. Your manuscript will remain under strict press embargo until 2 pm Eastern Time on the date of publication. For more information, please contact onepress@plos.org.

Kind regards,

Ahmed Abdelwahab Ibrahim El-Sayed, PhD, MSN in Nursing Administration, BSc in Nursing

Academic Editor

PLOS One

Additional Editor Comments (optional):

Dear Authors,

Thank you for submitting your manuscript and for your careful response to the reviewers’ comments.

I have now completed the final evaluation of the revised manuscript. The reviewers’ concerns have been adequately addressed, and the revisions have strengthened the manuscript considerably. The study is now presented with sufficient methodological clarity and scientific rigor, and its findings make a relevant contribution to the field.

Based on the revised manuscript and the reviewers’ recommendations, I am pleased to confirm that the manuscript is accepted for publication in PLOS ONE.

I congratulate the authors and thank you for choosing PLOS ONE to disseminate your work.

Reviewers' comments:

# Editor report: Acceptance letter

**DOI:** 10.1371/journal.pone.0356880.r008

**Authors:** Ahmed El-Sayed

PONE-D-26-11739R3

PLOS One

Dear Dr. Hsieh,

I'm pleased to inform you that your manuscript has been deemed suitable for publication in PLOS One. Congratulations! Your manuscript is now being handed over to our production team.

At this stage, our production department will prepare your paper for publication. This includes ensuring the following:

* All references, tables, and figures are properly cited

* All relevant supporting information is included in the manuscript submission,

* There are no issues that prevent the paper from being properly typeset

You will receive further instructions from the production team, including instructions on how to review your proof when it is ready. Please keep in mind that we are working through a large volume of accepted articles, so please give us a few days to review your paper and let you know the next and final steps.

Lastly, if your institution or institutions have a press office, please let them know about your upcoming paper now to help maximize its impact. If they'll be preparing press materials, please inform our press team within the next 48 hours. Your manuscript will remain under strict press embargo until 2 pm Eastern Time on the date of publication. For more information, please contact onepress@plos.org.

You will receive an invoice from PLOS for your publication fee after your manuscript has reached the completed accept phase. If you receive an email requesting payment before acceptance or for any other service, this may be a phishing scheme. Learn how to identify phishing emails and protect your accounts at https://explore.plos.org/phishing.

If we can help with anything else, please email us at customercare@plos.org.

Thank you for submitting your work to PLOS One and supporting open access.

Kind regards,

PLOS ONE Editorial Office Staff

on behalf of

Dr. Ahmed Abdelwahab Ibrahim El-Sayed

Academic Editor

PLOS One
